# ALK1 signaling is required for the homeostasis of Kupffer cells and prevention of bacterial infection

**DOI:** 10.1172/JCI150489

**Published:** 2022-02-01

**Authors:** Dianyuan Zhao, Fengjiao Yang, Yang Wang, Site Li, Yang Li, Fei Hou, Wenting Yang, Di Liu, Yuandong Tao, Qian Li, Jing Wang, Fuchu He, Li Tang

**Affiliations:** 1 State Key Laboratory of Proteomics, National Center for Protein Sciences, Beijing Proteome Research Center, Beijing Institute of Lifeomics, Beijing, China.; 2 Department of Immunology, School of Basic Medical Sciences, Anhui Medical University, Hefei, Anhui Province, China.; 3 Shanghai Institute of Immunology, Department of Immunology and Microbiology, Shanghai Jiao Tong University School of Medicine, Shanghai, China.

**Keywords:** Immunology, Macrophages

## Abstract

Macrophages are highly heterogeneous immune cells that fulfill tissue-specific functions. Tissue-derived signals play a critical role in determining macrophage heterogeneity. However, these signals remain largely unknown. The BMP receptor activin receptor–like kinase 1 (ALK1) is well known for its role in blood vessel formation; however, its role within the immune system has never been revealed to our knowledge. Here, we found that BMP9/BMP10/ALK1 signaling controlled the identity and self-renewal of Kupffer cells (KCs) through a Smad4-dependent pathway. In contrast, ALK1 was dispensable for the maintenance of macrophages located in the lung, kidney, spleen, and brain. Following ALK1 deletion, KCs were lost over time and were replaced by monocyte-derived macrophages. These hepatic macrophages showed significantly reduced expression of the complement receptor VSIG4 and alterations in immune zonation and morphology, which is important for the tissue-specialized function of KCs. Furthermore, we found that this signaling pathway was important for KC-mediated *Listeria monocytogenes* capture, as the loss of ALK1 and Smad4 led to a failure of bacterial capture and overwhelming disseminated infections. Thus, ALK1 signaling instructs a tissue-specific phenotype that allows KCs to protect the host from systemic bacterial dissemination.

## Graphical abstract

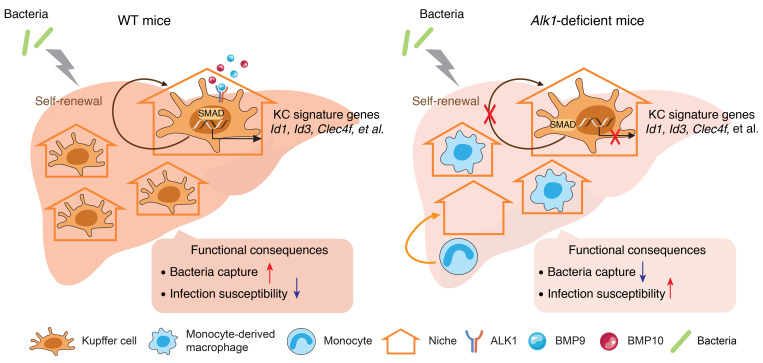

## Introduction

Kupffer cells (KCs) are resident macrophages in the liver and constitute 80%–90% of the tissue macrophages present in the body. They are predominantly derived from fetal liver monocytes (1–4). Embryonic KCs (em-KCs) are self-maintained and are not replaced by circulating monocytes under homeostatic conditions (1). However, if these em-KCs are deleted, blood monocytes also generate self-renewing KCs (5). Furthermore, regardless of their origin, embryonically derived and monocyte-derived KCs (Mo-KCs) have nearly identical transcriptional signatures, implying that shared signals control the specific transcriptional program of KCs (5). Recently, Bonnardel et al. and Sakai et al. proposed that during monocyte differentiation into KCs, these cells are sequentially programmed by signals derived from surrounding cells in the liver (6, 7). However, the signals provided by the liver niche that maintain the phenotype and survival of KCs remain to be investigated.

The liver is a primary site for the clearance of circulating bacteria, given its highly vascular architecture combined with a unique network of intravascular KCs (8–10). Unlike tissue-resident macrophages in other organs, KCs are strategically positioned in liver sinusoids and are directly exposed to slow-flowing sinusoidal blood, where they constantly trap and phagocytose circulating bacteria including gram-positive *Staphylococcus aureus* and *Listeria monocytogenes* (11–14), thus suggesting that KCs form an intravascular immune defense that prevents bacterial dissemination by capturing and clearing bacteria. However, little is known about the role of the liver environment in maintaining the integrity of KC-mediated intravascular defenses.

Activin receptor–like kinase 1 (ALK1, also known as ACVRL1) is a type I receptor of the TGFβ receptor superfamily with 2 ligands, BMP9 and BMP10 (15). ALK1 is predominantly expressed in endothelial cells and plays a critical role in regulating developmental and pathological angiogenesis (16). However, unlike TGFβR2 signaling, the role of ALK1 in the immune system has not been reported to date. Here, we found that BMP9/BMP10/ALK1 signaling controlled the specific gene expression program and survival of KCs through a Smad4-dependent pathway. Functionally, the loss of ALK1 resulted in impaired capture of *L. monocytogenes* and overwhelming disseminated infections. Taken together, our data reveal a previously unappreciated role of ALK1 signaling in maintaining KC homeostasis and function.

## Results

### Loss of Alk1, rather than of Tgfbr2, Alk2, or Alk3, leads to an altered phenotype of KCs.

Recently, Clec4F was identified as a specific surface marker for KCs (5). To specifically target KCs, we first generated *Clec4f^Cre/DTR^* mice (hereafter referred to as *Clec4f^Cre^*), in which an expression cassette encoding an internal ribosomal entry site (IRES), the Cre enzyme, a self-cleaving 2A peptide, and the human diphtheria toxin receptor (DTR) was inserted into the 3′-UTR of the *Clec4f* gene. We crossed *Clec4f^Cre^* mice with a conditional reporter strain (R26-tdTomato) to determine the efficiency and specificity of Cre-mediated recombination using flow cytometry. In the liver, the *Clec4f^Cre^* strain efficiently recombined in CD64^+^F4/80^+^ KCs (>90%), and almost all tdTomato^+^ cells were KCs (Supplemental Figure 1, A and B, and Supplemental Figure 2A; supplemental material available online with this article; https://doi.org/10.1172/JCI150489DS1). Furthermore, we did not detect tdTomato expression in other tissues (including CD45^+^ and CD45^–^ cells) (Supplemental Figure 1, C and D). Immunostaining experiments also confirmed that the reporter gene tdTomato was exclusively expressed in KCs and not in hepatocytes (Supplemental Figure 1E). In addition, 24 hours after diphtheria toxin (DT) administration, KCs were efficiently deleted in *Clec4f^Cre^* mice, as determined by immunostaining and flow cytometry (Supplemental Figure 1, F and G). Thus, the *Clec4f^Cre^* strain is a useful tool to specifically target KCs.

Recently, the expression of both the *Id1* and *Id3* genes was reported to be restricted to KCs compared with other tissue-resident macrophages, and Id3 deficiency impairs the differentiation of KCs (17). Loss of *Id3* results in reduced numbers of KCs (17). Interestingly, *Id1* is upregulated in *Id3*-deficient KCs, suggesting that Id1 may compensate for the function of Id3. The transcription factors *Id1* and *Id3* are target genes of BMP signaling (18), and thus an intriguing speculation is that BMPs present in the liver environment might be one of the tissue-derived signals that regulates KCs.

We first analyzed the expression of genes encoding BMP receptors and their coreceptors using data from the ImmGen Consortium to investigate which BMP signaling pathway regulates KCs and found that genes encoding BMPR2 and endoglin were expressed at high levels in KCs compared with expression levels in other tissue-resident macrophages (Supplemental Figure 3). Endoglin is required for BMP9/ALK1 signaling (19), and BMP9 is specifically expressed in the liver; therefore, a reasonable hypothesis is that ALK1 signaling might be important for KCs.

**Figure 1:**
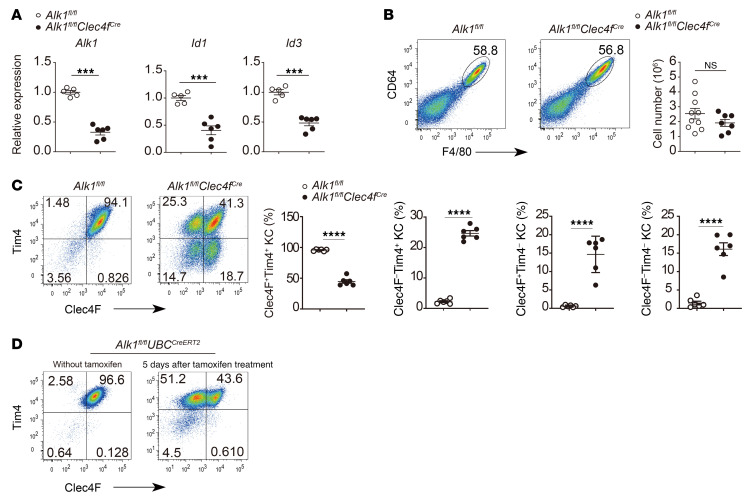
ALK1 controls the KC surface phenotype. (**A**) qPCR analysis of *Alk1*, *Id1*, and *Id3* expression in sorted KCs (CD45^+^Ly6C^–^CD64^+^F4/80^+^) from *Alk1^fl/fl^* *Clec4f^Cre^* mice and *Alk1^fl/fl^* controls (*n* = 5–6 per group). The gating strategy for KCs is shown in Supplemental Figure 2A. (**B**) Representative flow cytometric data and total number of CD64^+^F4/80^+^ KCs (pregated on CD45^+^Ly6C^–^) from *Alk1^fl/fl^* *Clec4f^Cre^* mice and *Alk1^fl/fl^* controls (*n* = 7–10 per group). (**C**) Flow cytometric expression of Clec4F and Tim4 in KCs (pregated on CD45^+^Ly6C^–^CD64^+^F4/80^+^) and the percentage of Clec4F^+^Tim4^+^, Clec4F^–^Tim4^+^, Clec4F^+^Tim4^–^, and Clec4F^–^Tim4^–^ KCs from *Alk1^fl/fl^* *Clec4f ^Cre^* mice and *Alk1^fl/fl^* control mice at the age of 8 weeks (*n* = 6 per group). (**D**) *Alk1^fl/fl^* *UBC^CreERT2^* mice were treated or not with tamoxifen (10 mg) 2 times every other day via oral gavage, and Clec4F and Tim4 expression in KCs was assessed 5 days after the last treatment. Data are representative of at least 3 independent experiments. Results represent the mean ± SEM. ***P < 0.001 and ****P < 0.0001, by 2-tailed Student’s *t* test (**A**–**C**).

We generated *Alk1^fl/fl^* *Clec4f^Cre^* mice to test this hypothesis. We also prepared *Alk2^fl/fl^* *Alk3^fl/fl^* *Clec4f^Cre^* mice. ALK1, ALK2, and ALK3 belong to the superfamily of TGFβ receptors, and TGFβ signaling has been proposed to be important for KCs (7). Thus, we also generated *Tgfbr2^fl/fl^* *Clec4f^Cre^* mice. *Alk2*, *Alk3*, and *Tgfbr2* were efficiently deleted in KCs from *Alk2^fl/fl^* *Alk3^fl/fl^* *Clec4f^Cre^* and *Tgfbr2^fl/fl^* *Clec4f^Cre^* mice, respectively, but the deficiency of these genes did not affect the expression of *Id1* and *Id3* (Supplemental Figure 4, A and B). In contrast, ablation of ALK1 resulted in a dramatic reduction in the expression of *Id1* and *Id3* in CD64^+^F4/80^+^ hepatic macrophages from *Alk1^fl/fl^* *Clec4f^Cre^* mice, although the expression of *Alk1* was reduced by approximately 60% in these macrophages (Figure 1A). We further analyzed the total hepatic macrophage population in *Alk1^fl/fl^* *Clec4f^Cre^* mice. We observed no difference in the number of KCs between *Alk1^fl/fl^* *Clec4f^Cre^* and *Alk1^fl/fl^* mice (Figure 1B), but the KC surface phenotype in *Alk1^fl/fl^* *Clec4f^Cre^* mice was altered, with a reduced population of Clec4F^+^Tim4^+^ KCs and increased populations of Clec4F^–^Tim4^+^ KCs, Clec4F^+^Tim4^–^ KCs, and Clec4F^–^Tim4^–^ KCs (Figure 1C). In addition, no difference was observed in the cell number and surface phenotype of KCs between *Alk2^fl/fl^* *Alk3^fl/fl^* *Clec4f^Cre^*, *Tgfbr2^fl/fl^* *Clec4f^Cre^,* and their littermate controls (Supplemental Figure 4, C–F). Taken together, these results suggested that ALK1, rather than ALK2, ALK3, and TGFβR2, is responsible for regulating the expression of *Id1* and *Id3* in KCs and plays an important role in maintaining the KC surface phenotype.

### ALK1 is required for the identity of KCs.

During homeostasis, nearly all KCs are Clec4F^+^Tim4^+^ cells. However, upon KC loss, newly arrived monocyte-derived KCs (MoKCs) are initially Clec4F^–^Tim4^–^ cells and then differentiate into Clec4F^+^Tim4^–^ cells (5). Among these Clec4F^+^Tim4^–^ MoKCs, only some acquire the expression of Tim4 (5). Thus, if KCs are constantly replaced by monocytes, 3 hepatic macrophage subsets are usually detected, based on the expression of Clec4F and Tim4, including Clec4F^–^Tim4^–^, Clec4F^+^Tim4^–^, and Clec4F^+^Tim4^+^ macrophages (20, 21). Interestingly, in addition to these macrophage subpopulations, we identified a population of Clec4F^–^Tim4^+^ KCs in *Alk1^fl/fl^* *Clec4f^Cre^* mice (Figure 1C) that, to our knowledge, has not been reported previously. Indeed, Clec4F^–^Tim4^+^ KCs were also detected in the livers of tamoxifen-treated *Alk1^fl/fl^* *UBC^CreERT2^* mice (Figure 1D), in which we assessed Clec4F and Tim4 expression 5 days after tamoxifen treatment. Thus, these results suggested that the presence of Clec4F^–^Tim4^+^ KCs may have been caused by *Alk1* loss.

**Figure 2:**
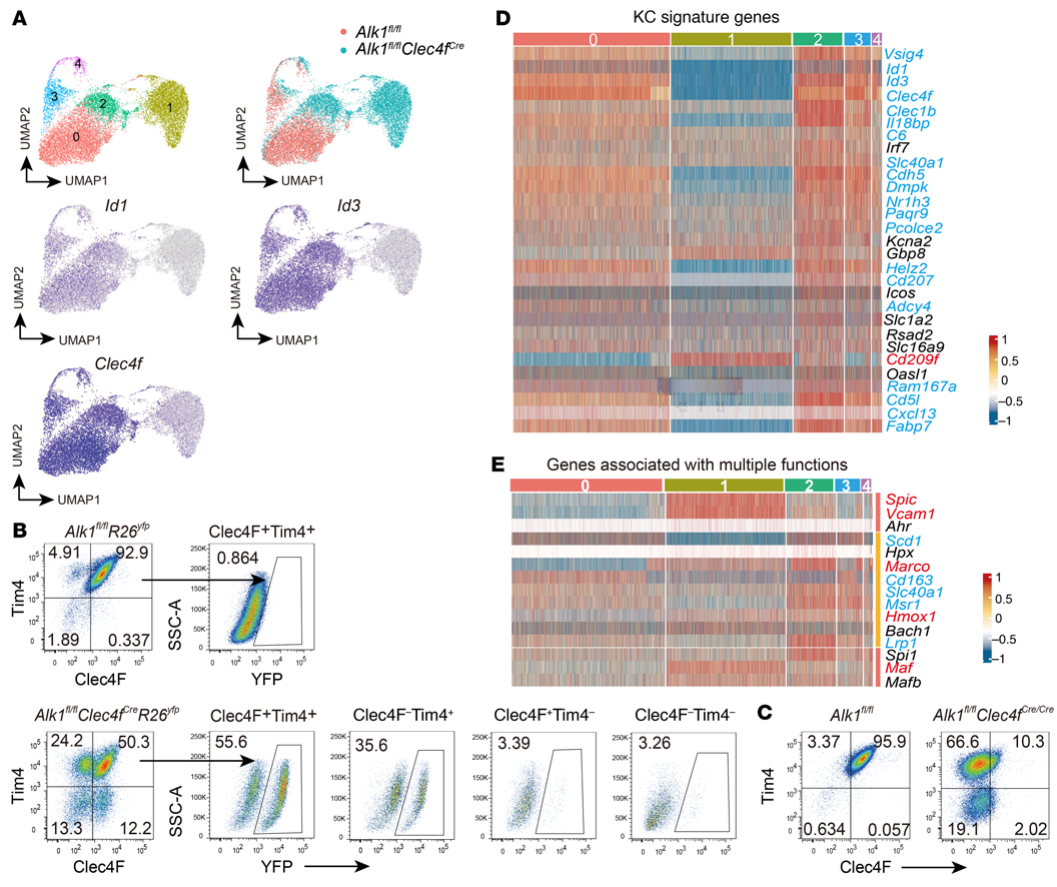
ALK1 controls KC identity. (**A**) UMAP plot of scRNA-Seq data on KCs from *Alk1^fl/fl^* *Clec4f ^Cre^* mice and *Alk1^fl/fl^* controls, showing clusters and the distribution of cells on different samples and expression of *Id1*, *Id3*, and *Clec4f* in KCs. (**B**) Flow cytometry of yellow fluorescent protein (YFP) in the indicated hepatic macrophage populations from *Alk1^fl/fl^* *Clec4f ^Cre^* *R26^yfp^* mice and *Alk1^fl/fl^* *R26^yfp^* controls. Data are representative of 3 independent experiments. (**C**) Expression of Clec4F and Tim4 in KCs (pregated on CD45^+^Ly6C^–^CD64^+^F4/80^+^) from *Alk1^fl/fl^* *Clec4f ^Cre/Cre^* mice and their controls at the age of 5 weeks. The experiment was repeated twice. (**D** and **E**) Heatmaps showing the indicated genes expressed differentially across KC clusters from scRNA-Seq data. Genes in red and blue were significantly upregulated and downregulated, respectively.

We next performed single-cell RNA-Seq (scRNA-Seq) analysis of sorted CD64^+^F4/80^+^Tim4^+^ KCs from *Alk1^fl/fl^* *Clec4f^Cre^* mice compared with their controls to understand how ALK1 affected KCs. After sequencing, aggregation of the samples, and removal of poor-quality and contaminating cells, 13,690 cells remained (6295 cells from *Alk1^fl/fl^* mice and 7395 cells from *Alk1^fl/fl^* *Clec4f^Cre^* mice). We identified 5 clusters by generating a uniform manifold approximation and projection (UMAP) from the transcriptome data using the Seurat pipeline (Figure 2A). Cluster 0 was predominantly composed of cells originating from *Alk1^fl/fl^* mice, whereas cells in clusters 1 and 2 mainly originated from *Alk1^fl/fl^* *Clec4f^Cre^* mice (Figure 2A). Because we were unable to determine which cells expressed full-length or floxed mRNA using the 3′ Assay from 10X Genomics, we next analyzed the differentially expressed (DE) genes between these clusters to find markers that could distinguish the different cell populations by flow cytometry. scRNA-Seq analysis revealed 243 DE genes in cluster 0, 347 DE genes in cluster 1, 182 DE genes in cluster 2, 189 DE genes in cluster 3, and 383 DE genes in cluster 4 (Supplemental Table 1) and showed that Clec4F was expressed in clusters 0, 2, 3, and 4, but not in cluster 1 (Figure 2A and Supplemental Figure 5A). Interestingly, we found that expression of *Id1* and *Id3* was also substantially reduced in cluster 1 (Figure 2A). Given that *Id1* and *Id3* are target genes of ALK1 signaling in KCs, these results suggested that Clec4F^–^Tim4^+^ KCs (cluster 1) may be deficient in *Alk1*. We performed quantitative PCR (qPCR) and genomic PCR on sorted Clec4F^–^Tim4^+^ and Clec4F^+^Tim4^+^ KCs from *Alk1^fl/fl^* *Clec4f^Cre^* mice to verify this result and found that Clec4F^–^Tim4^+^ KCs efficiently deleted *Alk1*, whereas Clec4F^+^Tim4^+^ KCs were heterozygous for the *Alk1* deletion (Supplemental Figure 5, B and C). KCs from *Alk1^fl/+^* *Clec4f^Cre^* mice did not display a phenotype similar to that of *Alk1^fl/fl^* *Clec4f^Cre^* mice (Supplemental Figure 5D), suggesting no obvious effect of *Alk1* haploinsufficiency on KCs.

In *Alk1^fl/fl^ Clec4f^Cre^* mice, Cre recombinase is expressed under the control of the Clec4F promoter, implying that Clec4F was once expressed in Clec4F^–^Tim4^+^ KCs. We prepared *Alk1^fl/fl^* *Clec4f^Cre^* *R26^yfp^* reporter mice to examine this possibility and observed high expression of the YFP reporter gene in Clec4F^–^Tim4^+^ KCs and Clec4F^+^Tim4^+^ KCs, but very low YFP expression in Tim4^–^ KCs (Figure 2B), suggesting that Clec4F^–^Tim4^+^ KCs once expressed Clec4F. Moreover, upon ALK1 deletion, KCs no longer expressed Clec4F. We further prepared *Alk1^fl/fl^* *Clec4f^Cre/Cre^* mice (homozygous for Cre) to increase the recombination frequency and to support this hypothesis and found that KCs from these mice did not express Clec4F (Figure 2C).

Based on the results described above, we identified cluster 1 as *Alk1^–/–^* KCs from *Alk1^fl/fl^* *Clec4f^Cre^* mice and cluster 0 as *Alk1^+/+^* KCs from *Alk1^fl/fl^* mice. Cells in cluster 2 from *Alk1^fl/fl^* *Clec4f^Cre^* mice were identified as *Alk1^+/–^* KCs. Clusters 3 and 4 were proliferating cells expressing DNA replication–associated genes such as *Mcm2-7* and *Mki67* (Supplemental Figure 5A). Then, we compared the transcriptional profiles between clusters and found that among the 25 top core genes of KCs described previously (20), the expression of 16 core genes was significantly reduced upon the loss of *Alk1* in KCs (Figure 2D), suggesting that ALK1 plays a critical role in maintaining the identity of KCs.

The transcription factors *Zeb2* and *Nr1h3* are required for the identity of KCs (20). We found that the expression of *Nr1h3*, but not *Zeb2*, was significantly decreased in *Alk1^–/–^* KCs (Figure 2D and Supplemental Table1). The transcription factor SPI-C is required for the development of splenic red pulp macrophages (RPMs), and its expression is induced by heme (22, 23). SPI-C expression was significantly upregulated in the absence of ALK1 (Figure 2E), but the CD163 and CD91 (encoded by *Lrp1*) receptors that uptake circulating hemoglobin-haptoglobin and heme-hemopexin complexes, respectively, were downregulated (Figure 2E). Based on these results, the ALK1 signaling pathway is required for KC identity and may negatively regulate *Spic* expression in KCs.

### The maintenance of KCs requires ALK1 signaling.

**Figure 3:**
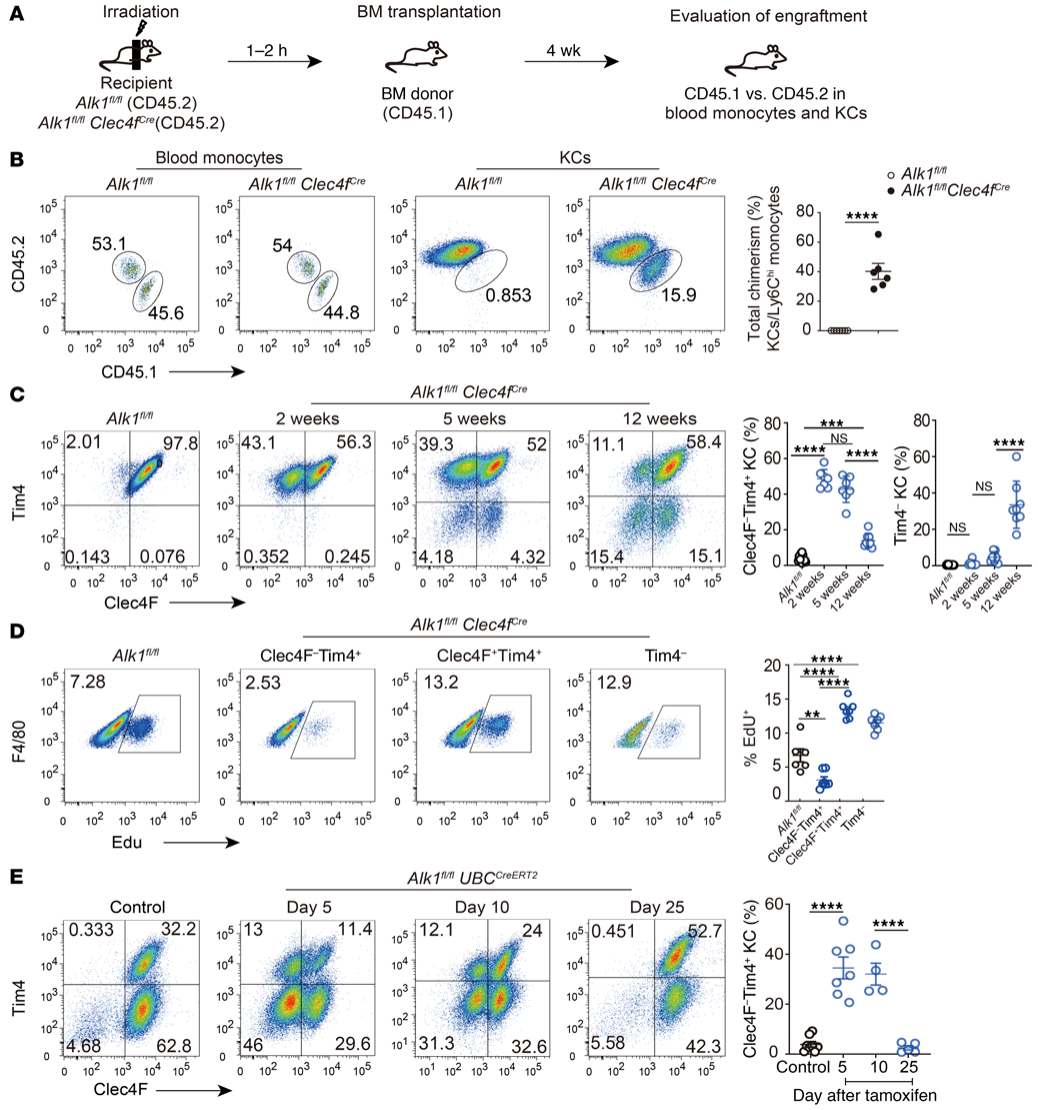
Maintenance of KCs requires ALK1 signaling. (**A**) Schematic of the experimental setup. (**B**) Expression of CD45.1 (donor) and CD45.2 (recipient) in blood monocytes and total KCs from *Alk1^fl/fl^* and *Alk1^fl/fl^* *Clec4f ^Cre^* chimeras. Plot shows the percentage of total chimerism of KCs in *Alk1^fl/fl^* and *Alk1^fl/fl^* *Clec4f ^Cre^* chimeras (*n* = 6 per group). The experimental setup was as indicated in **A**. (**C**) Expression of Clec4F and Tim4 and percentage of Clec4F^–^Tim4^+^ and Tim4^–^ cells in KCs from *Alk1^fl/fl^* *Clec4f ^Cre^* mice and *Alk1^fl/fl^* control mice at 2, 5, and 12 weeks of age (*n* = 7–12 per group). (**D**) Representative flow cytometric data and quantification of EdU incorporation in Clec4F^–^Tim4^+^, Clec4F^+^Tim4^+^, and Tim4^–^ KCs from *Alk1^fl/fl^* *Clec4f ^Cre^* mice and *Alk1^fl/fl^* controls (*n* = 6–7 per group). (**E**) The chimeric mice were treated with tamoxifen (10 mg) 2 times every other day via oral gavage. Clec4F and Tim4 expression in KCs originated from CD45.2^+^ donor cells was assessed 5, 10, and 25 days after the last treatment (*n* = 4–8 per group). Results represent the mean ± SEM. **P < 0.01, ***P < 0.001, and ****P < 0.0001, by 2-tailed Student’s *t* test (**B**) and 1-way ANOVA (**C**–**E**).

Decreased expression of Tim4 in KCs from *Alk1^fl/fl^* *Clec4f^Cre^* mice suggested that circulating monocytes might replenish liver macrophages. We generated shielded chimeras in which the livers of *Alk1^fl/fl^* *Clec4f^Cre^* mice and *Alk1^fl/fl^* littermate controls were shielded during irradiation, and these mice were reconstituted with congenic CD45.1 WT BM to examine this possibility (Figure 3A). As expected, partial shielding resulted in mixed chimerism in blood Ly6C^hi^ monocytes in all groups (Figure 3B). KCs in *Alk1^fl/fl^* mice were not chimeric, as KCs were self-maintained under steady-state conditions independent of circulating monocytes (Figure 3B). However, hepatic macrophages from *Alk1^fl/fl^* *Clec4f^Cre^* mice displayed chimerism (Figure 3B), suggesting that the ALK1 deficiency may have led to a loss of KCs and that circulating monocytes repopulated the empty niche to maintain the macrophage pool in the liver. Consistent with this result, the number of Clec4F^–^Tim4^+^ KCs decreased with age, and Tim4^–^ Mo-KCs expanded significantly over time (Figure 3C). 5-ethynyl-20-deoxyuridine (EdU) incorporation assays revealed that Clec4F^–^Tim4^+^ KCs had a reduced capacity to proliferate compared with their counterparts (Figure 3D), indicating that a decrease in the proliferation of *Alk1*-deficient KCs leads to a severe disadvantage of these cells under competitive conditions. Maf and Mafb function as negative regulators of KC proliferation (24). Consistent with the impaired proliferation, we found that *Maf* was expressed at higher levels in *Alk1*-deficient KCs than in their counterparts (Figure 2E). Thus, these results suggested that ALK1 may be required for the maintenance of KCs.

To determine whether ALK1 deficiency results in KC disappearance, we established BM chimeras in which CD45.1^+^ mice were lethally irradiated and injected with congenic CD45.2^+^ *Alk1^fl/fl^* *UBC^CreERT2^* BM, in which tamoxifen administration leads to deletion of *Alk1* in a wide range of cells (25). The chimeric mice were treated with tamoxifen 8 weeks after reconstitution. Based on a previous report (5), Mo-KCs are able to differentiate into mature Clec4F^+^ KCs, but only some of these cells acquire Tim4 expression. Our results also confirmed this finding (Figure 3E). Consistent with the aforementioned observation that ALK1 is important for Clec4F expression, we observed reduced expression of Clec4F in Tim4^+^ and Tim4^–^ KCs after tamoxifen administration (Figure 3E). Because a good antibody is unavailable to stain ALK1 for flow cytometry and we cannot exclude the possibility that Clec4F^–^Tim4^–^ KCs were derived from newly arrived WT MoKCs, we used Clec4F^–^Tim4^+^ KCs to represent *Alk1*-deficient cells and determined Clec4F expression in Tim4^+^ KCs originating from CD45.2^+^ donor cells 5, 10, and 25 days after the last treatment with tamoxifen. Chimeras not treated with tamoxifen were used as controls. Approximately 30% of donor-derived Tim4^+^ KCs were Clec4F^–^ on days 5 and 10 after the last treatment, whereas Clec4F^–^Tim4^+^ KCs were no longer detected in the liver 25 days after the last treatment (Figure 3E), indicating that *Alk1*-deficient KCs were lost over time.

### ALK1 is dispensable for the maintenance of macrophages located in the lung, kidney, brain, and spleen.

**Figure 4:**
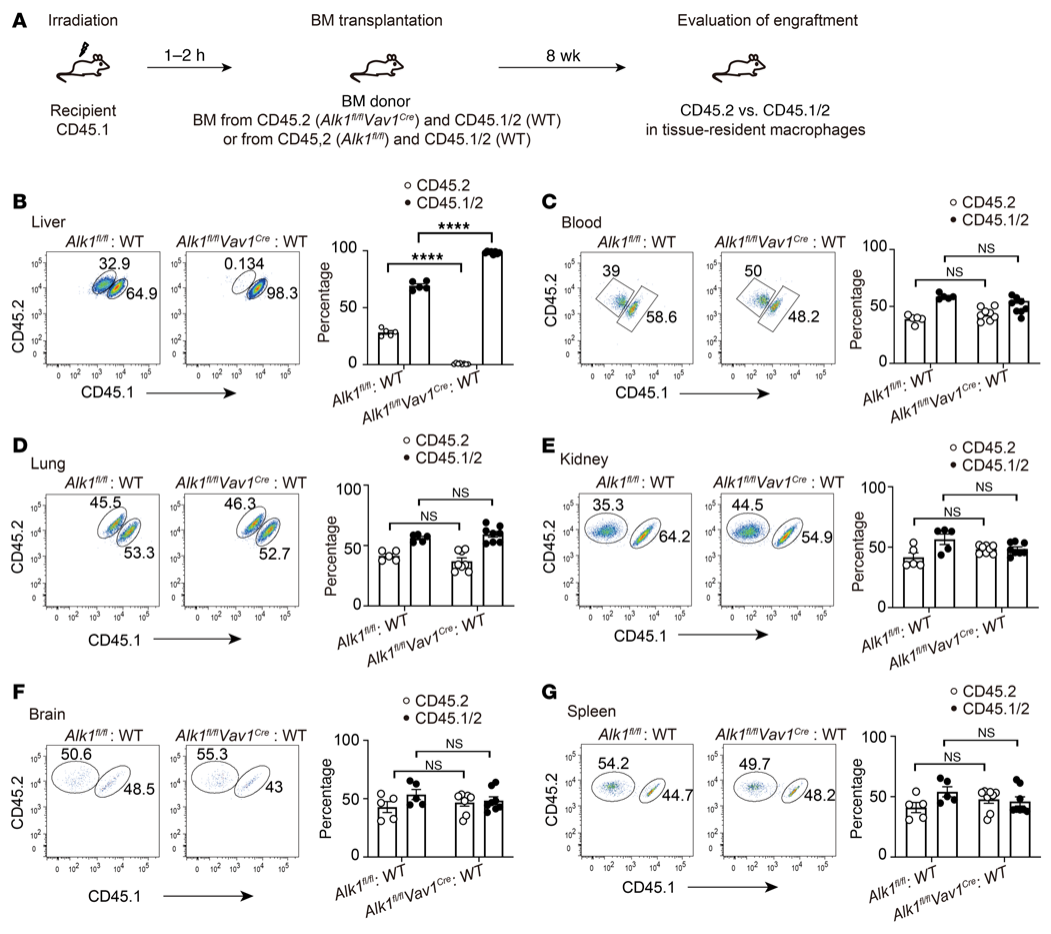
ALK1 is dispensable for the maintenance of macrophages located in the lung, kidney, brain, and spleen. (**A**) Schematic of the experimental setup. (**B**–**G**) Representative flow cytometric data and percentages of CD45.1^+^CD45.2^+^ and CD45.2^+^ of KCs (**B**), blood monocytes (**C**), alveolar macrophages (AMs) (**D**), kidney macrophages (**E**), brain macrophages (**F**), and splenic macrophages (**G**). *n* = 5–8 per group. Data were pooled from 2 independent experiments. Results represent the mean ± SEM. ****P < 0.0001, by 2-way ANOVA. Gating strategies for the indicated macrophages in **B**–**G** are shown in Supplemental Figure 2.

To assess whether ALK1 signaling is required for the maintenance of other tissue-resident macrophages, we generated mixed BM chimeras. We used *Alk1^fl/fl^Vav1^Cre^* mice, in which Cre recombinase is expressed at high levels in hematopoietic stem cells and maintains robust activity during reconstitution following transplantation (26). Recipient mice (CD45.1) were lethally irradiated and reconstituted with equal amounts of WT (CD45.1/CD45.2) and *Alk1^fl/fl^* *Vav1^Cre^* (CD45.2) BM or WT (CD45.1/CD45.2) and *Alk1^fl/fl^* (CD45.2) BM (Figure 4A). After 8 weeks, we examined the origin of KCs. In this competitive setting, approximately all KCs originated from WT (CD45.1/CD45.2) BM in WT (CD45.1/CD45.2) and *Alk1^fl/fl^* *Vav1^Cre^* (CD45.2) chimeras compared with that seen in WT (CD45.1/CD45.2) and *Alk1^fl/fl^* (CD45.2) chimeras (Figure 4B), suggesting that liver macrophages deficient in *Alk1* are outcompeted by their WT counterparts. In contrast, blood monocytes and other tissue macrophages, including lung macrophages, kidney macrophages, brain macrophages, and splenic macrophages, were reconstituted at an equal ratio in WT (CD45.1/CD45.2) and *Alk1^fl/fl^* *Vav1^Cre^* (CD45.2) chimeras (Figure 4, C–G). Taken together, these results suggested that ALK1 is dispensable for the survival of macrophages located in the lung, brain, kidney, and spleen.

### BMP9 and BMP10 instruct KC signature gene expression.

**Figure 5:**
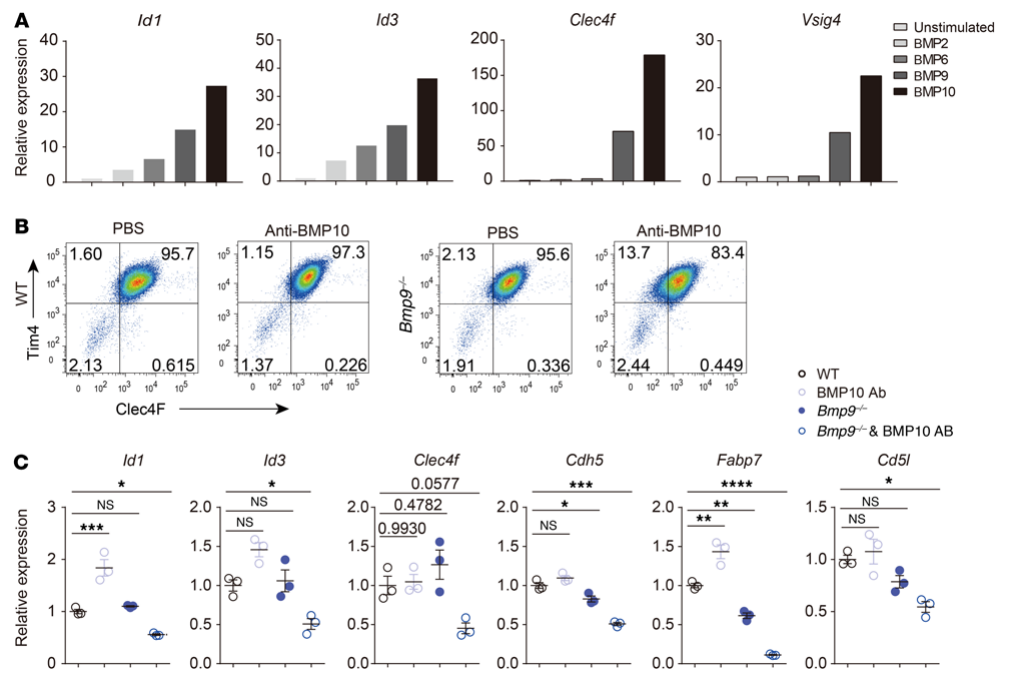
BMP9 and BMP10 control the expression of the KC-specific signature gene. (**A**) Sorted KCs were stimulated with BMP2, BMP6, BMP9, or BMP10, and expression of the indicated genes was determined by qPCR. Data are representative of at least 3 independent experiments. (**B**) Flow cytometric analysis of Clec4F and Tim4 in KCs from *Bmp9*-KO and WT mice treated i.p. with PBS or anti-BMP10 antibody (15 mg/kg) 4 times every day. Data are representative of 3 independent experiments. (**C**) KCs were sorted from the mice described as in **B**, and expression of the indicated genes was determined by qPCR (*n* = 3 per group). Results represent the mean ± SEM. *P < 0.05, **P < 0.01, ***P < 0.001, and ****P < 0.0001, by 1-way ANOVA (**C**).

Both BMP9 and BMP10 are ligands of ALK1. In the results described above, we showed that ALK1, rather than ALK2 and ALK3, was required for the expression of *Id1*, *Id3*, and *Clec4f*. Consistent with these results, BMP9 and BMP10 treatment maintained higher expression of *Id1*, *Id3*, and *Clec4f* in cultured KCs than did BMP2 (ALK3 ligand) or BMP6 (ALK2 ligand) (Figure 5A). However, Clec4F expression in KCs from *Bmp9*-KO mice was unaltered compared with that in KCs from WT littermate controls (Figure 5B). In fact, it has been reported that BMP10 is able to compensate for the loss of BMP9 (27, 28). To verify this, we administered an anti-BMP10 neutralizing antibody to *Bmp9*-KO mice and found that it resulted in decreased expression of Clec4F, whereas an injection of this antibody into WT mice did not affect its expression (Figure 5B). qPCR analysis revealed that KC-specific genes, such as *Id1*, *Id3*, *Clec4f*, *Fabp7*, *Cd5l*, and *Cdh5*, were most significantly downregulated in KCs from *Bmp9*-KO mice treated with the anti-BMP10 antibody compared with their controls (Figure 5C). Thus, both BMP9 and BMP10 are important for maintaining KC identity.

### ALK1 signaling functions in KCs through the canonical Smad pathway.

**Figure 6:**
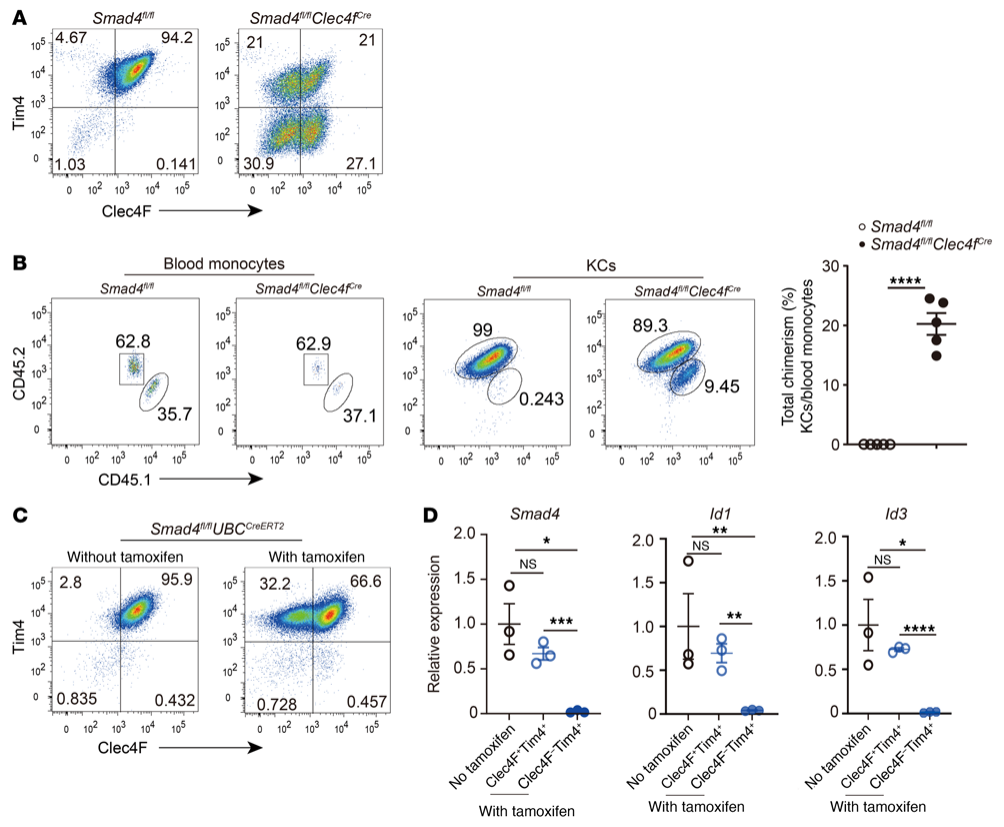
Loss of Smad4 in KCs repeats the altered phenotype of loss of ALK1. (**A**) Expression of Clec4F and Tim4 in KCs (pregated on CD45^+^Ly6C^–^CD64^+^F4/80^+^) in *Smad4^fl/fl^* *Clec4f^Cre^* mice and their controls. Data are representative of at least 3 independent experiments. (**B**) Expression of CD45.1 (donor) and CD45.2 (recipient) in blood monocytes and total KCs in *Smad4^fl/fl^* *Clec4f^Cre^* and *Smad4^fl/fl^* chimeras. Plot shows the percentage of total chimerism of KCs in *Smad4^fl/fl^* *Clec4f^Cre^* and *Smad4^fl/fl^* chimeras (*n* = 5 per group). (**C**) *Smad4^fl/fl^* *UBC^CreERT2^* mice were treated or not with tamoxifen (10 mg) 2 times every other day via oral gavage, and Clec4F and Tim4 expression in KCs (pregated on CD45^+^Ly6C^–^CD64^+^F4/80^+^) was assessed 5 days after the last treatment. Data are representative of at least 3 independent experiments. (**D**) qPCR analysis of *Smad4*, *Id1*, and *Id3* expression in sorted total KCs, Clec4F^+^Tim4^+^ KCs, and Clec4F^–^Tim4^+^ KCs from the mice described in **C** (*n* = 3 per group). Results represent the mean ± SEM. *P < 0.05, **P < 0.01, ***P < 0.001, and ****P < 0.0001, by 2-tailed Student’s *t* test (**B**) and 1-way ANOVA (**D**).

Smad4 functions as a common Smad required for transcriptional regulation in response to BMPs. We generated *Smad4^fl/fl^* *Clec4f^Cre^* mice to assess whether ALK1 signaling functions in KCs via the Smad pathway. Similar to the effect of *Alk1* deletion, KCs from *Smad4^fl/fl^* *Clec4f^Cre^* mice displayed altered expression of Clec4F and Tim4 (Figure 6A). In protected chimeras, Smad4 deficiency led to the replenishment of KCs from monocytes (Figure 6B). Similar to our findings in *Alk1^fl/fl^* *UBC^CreERT2^* mice, Clec4F^–^Tim4^+^ cells were also detected in KCs from tamoxifen-treated *Smad4^fl/fl^* *UBC^CreERT2^* mice (Figure 6C). We sorted Clec4F^–^Tim4^+^ and Clec4F^+^Tim4^+^ KCs and examined the expression of *Smad4*, *Id1*, and *Id3*. qPCR analysis revealed that Clec4F^–^Tim4^+^ KCs had efficiently deleted *Smad4*, whereas Clec4F^+^Tim4^+^ KCs maintained the expression of *Smad4* at levels comparable to those in KCs isolated from untreated *Smad4^fl/fl^* *UBC^CreERT2^* mice (Figure 6D). Accordingly, its target genes, *Id1* and *Id3*, were significantly reduced in Clec4F^–^Tim4^+^ KCs but not in Clec4F^+^Tim4^+^ KCs. In summary, we found that ALK1 signaling regulated KCs through the canonical Smad pathway.

### The functional phenotype of KCs is maintained by ALK1 signaling.

To investigate the functional consequences of ALK1 deletion in KCs, we generated ALK1 conditional-KO mice, in which *Alk1* was completely targeted in these cells. *Alk1* was not efficiently deleted in KCs from *Alk1^fl/fl^* *Clec4f^Cre^* mice, so we analyzed *Alk1^fl/fl^* *Vav1^Cre^* mice. qPCR analysis revealed that *Alk1* was successfully deleted in the total population of CD64^+^F4/80^+^ hepatic macrophages (Supplemental Figure 6A). Moreover, *Id1* and *Id3* expression was significantly reduced in KCs from *Alk1^fl/fl^* *Vav1^Cre^* mice compared with those from *Alk1^fl/fl^* mice (Supplemental Figure 6A), and Clec4F expression was not detected in either Tim4^+^ or Tim4^–^ KCs from *Alk1^fl/fl^* *Vav1^Cre^* mice (Supplemental Figure 6B). We also analyzed the number of hepatic myeloid cells, including KCs, neutrophils, monocytes, plasmacytoid DCs (pDCs), and conventional DCs (cDCs), and found that these cell counts were normal in *Alk1^fl/fl^* *Vav1^Cre^* mice (Supplemental Figure 6, C–F). Similarly, *Smad4* was also efficiently targeted in KCs from *Smad4^fl/fl^* *Vav1^Cre^* mice (Supplemental Figure 6, G and H).

In addition, we examined E-cadherin and glutamine synthetase expression and interactions between KCs, liver sinusoidal endothelial cells (LSECs), and hepatic stellate cells (HSCs) in *Alk1^fl/fl^* *Vav1^Cre^* and *Smad4^fl/fl^* *Vav1^Cre^* mice to determine whether the liver architecture was affected by ALK1 and Smad4 deficiency. We found that E-cadherin and glutamine synthetase were expressed in the periportal and central vein regions of the liver lobules, respectively. The regional localization of E-cadherin and glutamine synthetase in the liver lobules of *Alk1^fl/fl^* *Vav1^Cre^* and *Smad4^fl/fl^* *Vav1^Cre^* mice was not affected (Supplemental Figure 7, A and B), and KCs from these mice still closely interacted with LSECs and HSCs (Supplemental Figure 7, C and D), suggesting that the liver architecture was intact.

**Figure 7:**
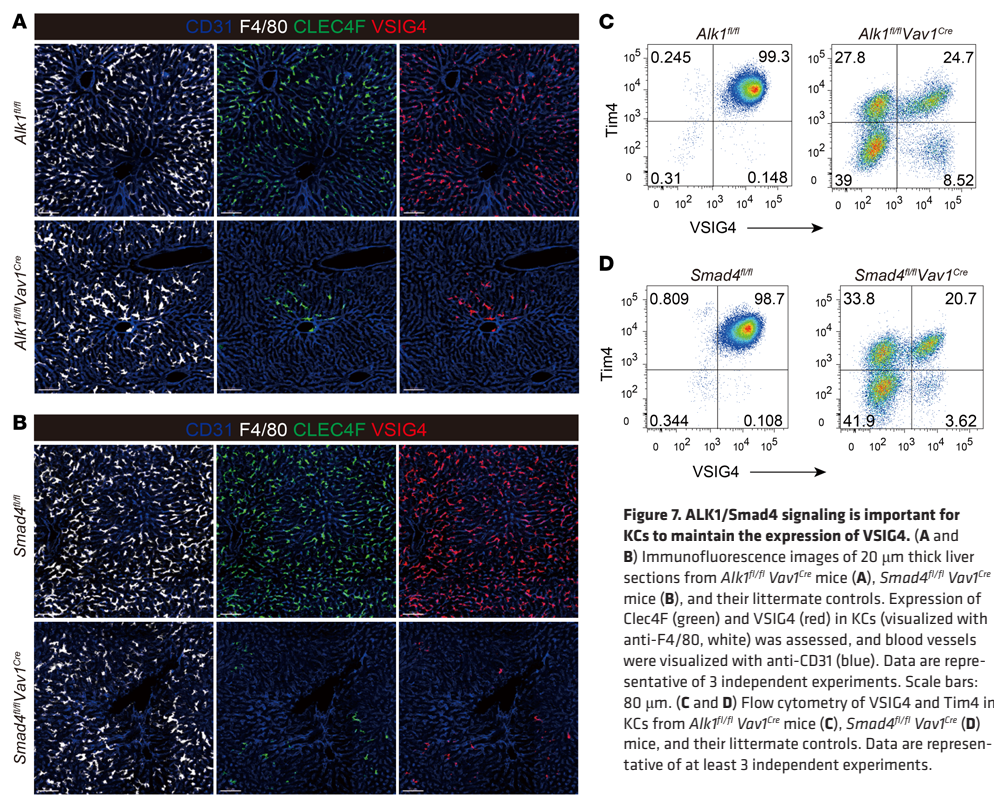
ALK1/Smad4 signaling is important for KCs to maintain the expression of VSIG4. (**A** and **B**) Immunofluorescence images of 20 μm thick liver sections from *Alk1^fl/fl^* *Vav1^Cre^* mice (**A**), *Smad4^fl/fl^* *Vav1^Cre^* mice (**B**), and their littermate controls. Expression of Clec4F (green) and VSIG4 (red) in KCs (visualized with anti-F4/80, white) was assessed, and blood vessels were visualized with anti-CD31 (blue). Data are representative of 3 independent experiments. Scale bars: 80 μm. (**C** and **D**) Flow cytometry of VSIG4 and Tim4 in KCs from *Alk1^fl/fl^* *Vav1^Cre^* mice (**C**), *Smad4^fl/fl^* *Vav1^Cre^* (**D**) mice, and their littermate controls. Data are representative of at least 3 independent experiments.

VSIG4 (also known as CRIg), a new family of complement receptors, was reported to be expressed by KCs at high levels (13) and plays an important role in KC-mediated capture of gram-positive bacteria (11, 13, 14). qPCR analysis revealed that *Vsig4* in KCs was obviously upregulated after BMP9 and BMP10 treatment (Figure 5A). The transcriptomic analysis revealed that *Vsig4* expression was significantly decreased following the deletion of *Alk1* in KCs (Figure 2D). Confocal microscopy and flow cytometry also confirmed this significant reduction in KCs from *Alk1^fl/fl^* *Vav1^Cre^* and *Smad4^fl/fl^* *Vav1^Cre^* mice (Figure 7, A–D).

**Figure 8:**
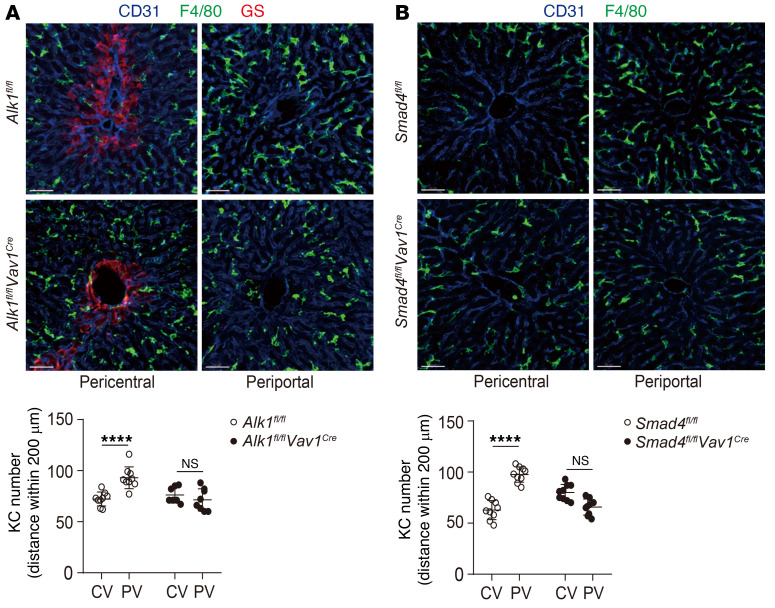
ALK1/Smad4 signaling is important for KCs to maintain their location. (**A** and **B**) Zoom-in representative immunofluorescence images of liver sections from *Alk1^fl/fl^* *Vav1^Cre^* mice (**A**), *Smad4^fl/fl^* *Vav1^Cre^* mice (**B**), and their littermate controls showing the distribution of KCs (anti-F4/80, green) at the periphery of CVs (visualized with anti–glutamine synthetase, red) and PVs. Blood vessels were visualized with anti-CD31 (blue). KC numbers in each region of 200 μm radius around CVs or PVs were quantified. Scale bars: 50 μm. Data were pooled from 3 mice per group. Results represent the mean ± SEM. ****P < 0.0001, by 2-way ANOVA (**A** and **B**).

**Figure 9:**
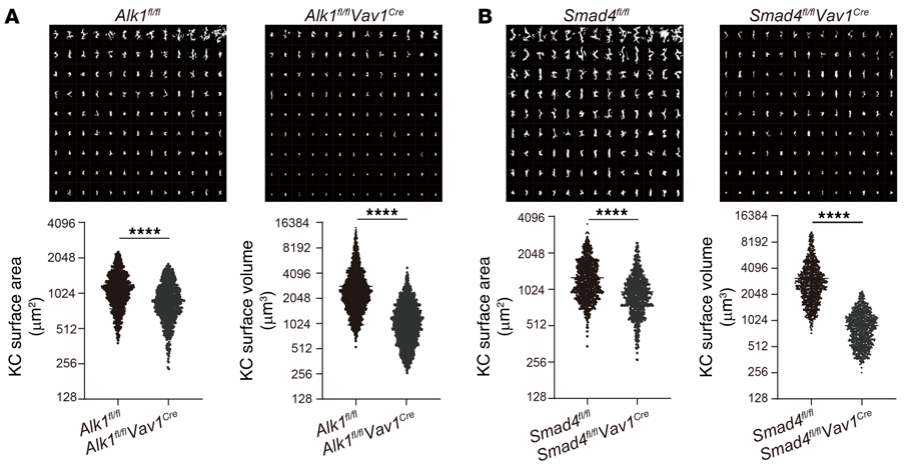
ALK1/Smad4 signaling is important for KCs to maintain their morphology. (**A** and **B**) Rendered surfaces (*n* = 117) of KCs from *Alk1^fl/fl^* *Vav1^Cre^* mice (**A**), *Smad4^fl/fl^* *Vav1^Cre^* mice (**B**), and their littermate controls were displayed and arranged by surface area (top) and 3D analysis of the surface area and surface volume of individual KCs (bottom). Data were pooled from 3 mice per group. Results represent the mean ± SEM. ****P < 0.0001, by 2-tailed Student’s *t* test (**A** and **B**).

In addition to the cell surface phenotype, KC function is also governed by the cell’s 3D morphology within the vasculature, as its elongated and branched shape increases the intravascular surface area available for interacting with circulating pathogens (9). KCs are strategically enriched near periportal regions. This positional asymmetry (immune zonation) is important for KCs to protect against the systemic dissemination of pathogens from local infection sites such as the digestive tract (29). Interestingly, *Alk1^fl/fl^* *Vav1^Cre^* and *Smad4^fl/fl^* *Vav1^Cre^* mice lacked the periportal polarization of KCs observed in their controls (Figure 8, A and B). In addition, KCs from *Alk1^fl/fl^* *Vav1^Cre^* and *Smad4^fl/fl^* *Vav1^Cre^* mice were generally smaller, with a decreased cell surface area and volume compared with KCs from *Alk1^fl/fl^* control mice (Figure 9, A and B). Taken together, ALK1 signaling plays an important role in maintaining the functional phenotype of KCs.

### The ALK1 signaling pathway in KCs protects the host from infection with L. monocytogenes.

**Figure 10:**
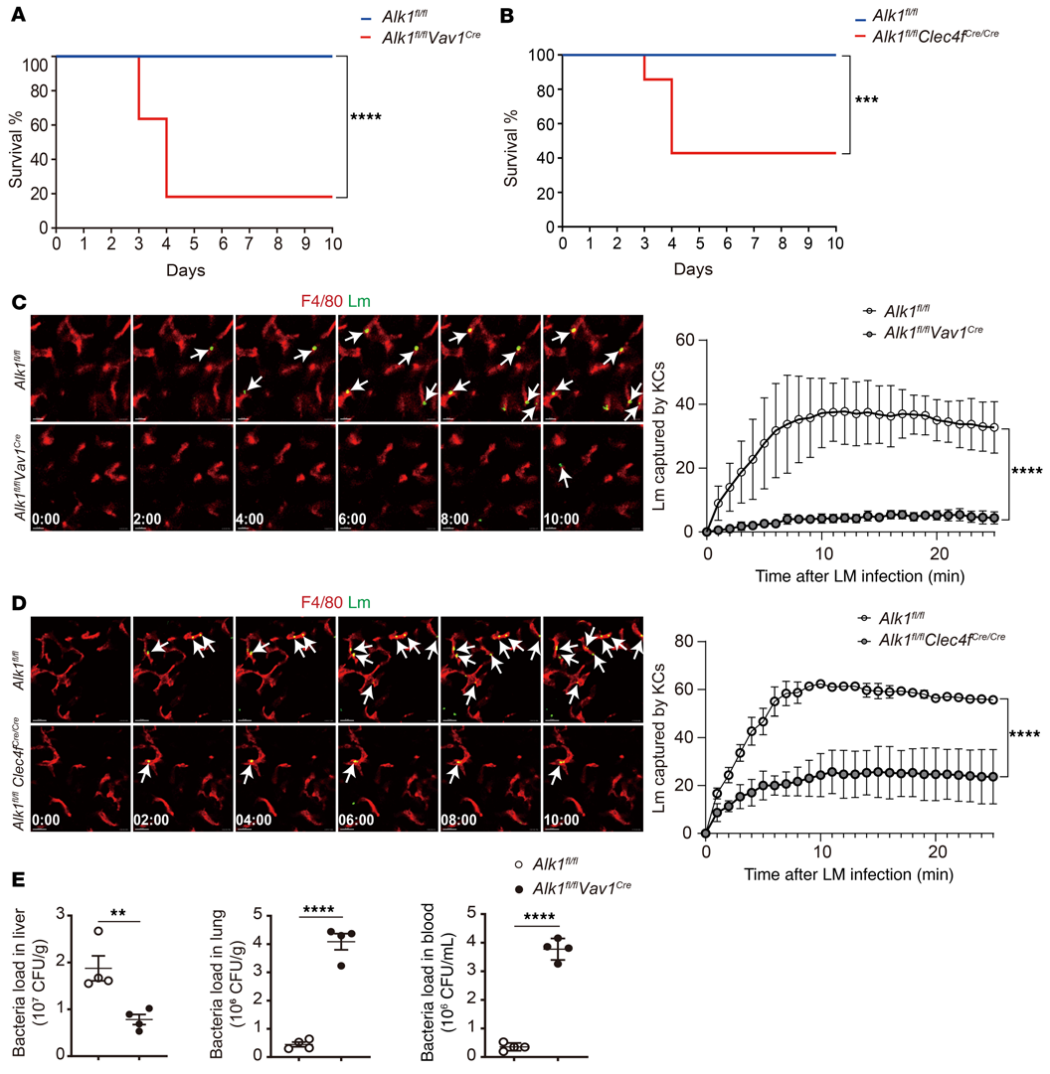
*Alk1*-deficient mice exhibit increased susceptibility to *L. monocytogenes* infection. (**A** and **B**) Survival of *Alk1^fl/fl^* *Vav1^Cre^* mice (**A**, *n* = 11), *Alk1^fl/fl^* *Clec4f^Cre/Cre^* mice (**B**, *n* = 7), and their littermate controls (*n* = 8–14 mice per group) infected with 5 × 10^5^ CFU *L. monocytogenes* was monitored daily. (**C** and **D**) Representative IVM images showing KCs (anti-F4/80, red) capturing circulating *L. monocytogenes* (Lm) (CFSE, green) within 25 minutes of infection in *Alk1^fl/fl^* *Vav1^Cre^* mice (**C**, *n* = 3), *Alk1^fl/fl^* *Clec4f^Cre/Cre^* mice (**D**, *n* = 3), and their littermate controls (*n* = 3–4 mice per group). Bacteria captured by KCs are highlighted by arrows. Scale bars: 10 μm. Quantification of captured *L. monocytogenes* is shown in the graphs on the right. (**E**) CFU were assayed in liver, lung, and blood of *Alk1^fl/fl^* *Vav1^Cre^* mice and their littermate controls 10 minutes after injection of *L. monocytogenes* (4 × 10^7^ CFU, *n* = 4 per group). The experiment was repeated twice. Results represent the mean ± SEM. **P < 0.01, ***P < 0.001, and ****P < 0.0001, by Mantel-Cox test (**A** and **B**), 2-way ANOVA (**C** and **D**), and 2-tailed Student’s *t* test (**E**).

Next, we infected *Alk1^fl/fl^* *Vav1^Cre^* and *Alk1^fl/fl^* mice with *L. monocytogenes* and found that approximately 80% of *Alk1^fl/fl^* *Vav1^Cre^* mice died within 7 days of infection, whereas no *Alk1^fl/fl^* mice succumbed to the infection (Figure 10A). Similarly, *Smad4^fl/fl^* *Vav1^Cre^* mice exhibited higher mortality following *L. monocytogenes* infection than did *Smad4^fl/fl^* control mice (Supplemental Figure 8A). Vav1-Cre not only deletes floxed genes in KCs, but also in other cell types, such as hematopoietic stem cells and their progenies. To examine whether the influence of ALK1 on controlling *L. monocytogenes* infection is KC intrinsic, we infected *Alk1^fl/fl^* *Clec4f^Cre/Cre^* and *Alk1^fl/fl^* mice with *L. monocytogenes* and found that *Alk1^fl/fl^Clec4f^Cre/Cre^* mice were more susceptible to *L. monocytogenes* infection than were their controls (Figure 10B), suggesting that ALK1 has a KC-intrinsic role in protecting the host from *L. monocytogenes* infection. Moreover, we used intravital microscopy (IVM) to visualize and quantify the bacterial capture within the liver and to understand the mechanism underlying the increased susceptibility to infection and found that *Alk1/Smad4*-deficient mice showed a significant reduction in the capture of circulating *L. monocytogenes* by KCs (Figure 10, C and D, Supplemental Figure 8B, and Supplemental Videos 1–3), despite having a similar number of KCs compared with their controls (Supplemental Figure 7, E and F). As a result, rapid systemic bacterial dissemination occurred in *Alk1^fl/fl^* *Vav1^Cre^* mice, with less *L. monocytogenes* in the liver but significantly more bacteria in the lung and blood (Figure 10E). These results show that the ability of KCs to capture bacteria from the bloodstream was significantly impaired in *Alk1^fl/fl^* *Vav1^Cre^* mice, resulting in systemic bacterial dissemination and increased mortality of the host.

KCs are essential for host survival in *L. monocytogenes* infection, but most *L. monocytogenes* are not killed by the KCs (30). Elimination of the pathogen in *L. monocytogenes* infection requires the recruitment of neutrophils to the liver and the specific binding of these neutrophils to KCs (30). We found that neutrophil recruitment to the liver occurred 2 hours after infection, and we observed no difference in their recruitment and interaction with KCs between *Alk1^fl/fl^ Vav1^Cre^* and *Alk1^fl/fl^* mice (Supplemental Figure 9).

IFN-γ is essential for the innate defense against *L. monocytogenes* infection (31). We observed increased expression of intracellular IFN-γ in NK cells, CD4^+^ T cells, and CD8^+^ T cells from *Alk1^fl/fl^* *Vav1^Cre^* mice 24 hours after infection (Supplemental Figure 10A), which coincided with higher bacterial counts in the livers and spleens of *Alk1^fl/fl^Vav1^Cre^* mice than in *Alk1^fl/fl^* mice (Supplemental Figure 10B). Recently, it has been shown that IL-17A plays a critical role in innate defense against *L. monocytogenes* infection in the liver (32). Consistent with the previous report (32), we found that IL-17A was mainly expressed by TCR γδ^+^ T rather than CD4^+^ T cells 5 days after *L. monocytogenes* infection (Supplemental Figure 10C). The proportion of IL-17A–producing cells in TCR γδ^+^ T cells in the liver of *Alk1^fl/fl^* *Vav1^Cre^* mice was comparable to that in the liver of *Alk1^fl/fl^* mice (Supplemental Figure 10C), indicating that the ability of TCR γδ^+^ T cells to produce IL-17A was not impaired in *Alk1^fl/fl^* *Vav1^Cre^* mice.

To investigate whether KC phagosome maturation and activation are altered in the absence of ALK1, we quantified phagocytosis and ROS production by KCs that engulfed *L. monocytogenes* in vivo using pH- and ROS-sensitive probe-labeled bacteria. We observed no differences between *Alk1^fl/fl^* *Vav1^Cre^* and *Alk1^fl/fl^* mice in the acidification or ROS production of *L. monocytogenes*–containing phagosomes in KCs (Supplemental Figure 11, A and B). Furthermore, we also determined cytokine expression by KCs from *Alk1^fl/fl^ Vav1^Cre^* and *Alk1^fl/fl^* mice in response to *L. monocytogenes* infection and found that expression of the TNF-α and MCP-1 mRNAs in KCs was slightly reduced in the absence of ALK1 (Supplemental Figure 11C), indicating decreased KC activation in *Alk1^fl/fl^* *Vav1^Cre^* mice during *L. monocytogenes* infection. Overall, these results suggested that the ALK1 signaling pathway is critical for host defenses against *L. monocytogenes* infection and that this effect appears to be KC specific.

## Discussion

Most tissue-resident macrophages were derived from yolk sac macrophages or fetal liver monocytes. Once progenitors arrive at their tissue of residence, they undergo extensive differentiation according to molecular cues provided by their tissue-specific niche (33). This process enables these progenitors to develop into specialized tissue-resident macrophages with a unique transcription profile. However, the precise signals governing this process remain largely unknown. Here, we identified a critical role for BMP9/BMP10/ALK1 signaling in imprinting KC identity. Loss of ALK1 impaired the ability of KCs to proliferate. Notably, we found that ALK1 was dispensable for the survival of macrophages in many organs, which demonstrated a specific role in the maintenance of KCs.

Clec4F is selectively expressed in mature KCs. In contrast to F4/80, which is a constitutively expressed surface marker for resident macrophages (34, 35), Clec4F is inducible in the liver microenvironment (36). Embryonic progenitors and monocytes progressively acquire Clec4F expression upon entry into the liver (5, 36). Thus, studies aiming to understand what types of signals or molecules are necessary to regulate Clec4F expression in the liver microenvironment would be interesting (36). We and other researchers (6) showed that BMP9 stimulation induces the expression of *Clec4f*. In the present study, in vivo blockade of ALK1 signaling by injecting anti-BMP10–blocking antibody into *Bmp9*-KO mice resulted in decreased expression of Clec4Fat mRNA and protein levels. Moreover, KCs no longer expressed Clec4F when *Alk1* was completely deleted, as observed in KCs from *Alk1^fl/fl^* *Vav1^Cre^* and *Alk1^fl/fl^* *Clec4f^Cre/Cre^* mice. Together, these results suggested that ALK1 signaling is essential for Clec4F expression in KCs.

BMP9 is preferentially expressed in the liver. In the present study, in addition to BMP9, BMP10 was critical for controlling the identity of KCs. BMP10 is mainly expressed in the heart and present in blood (27). However, this circulating BMP10 is unable to activate the ALK1 signaling pathway (27). In fact, it is also weakly expressed in the liver (37). The source of BMP9 and BMP10 was reported to be HSCs (38, 39), indicative of a paracrine loop that regulates KC identity and self-maintenance.

KCs are enriched near periportal regions. This asymmetric localization also has a critical role in protecting against systemic bacterial dissemination (29). Interestingly, we found that hepatic macrophages lost their tissue-specific localization in *Alk1^fl/fl^* *Vav1^Cre^* mice and *Smad4^fl/fl^* *Vav1^Cre^* mice. CXCR3 expressed on KCs has been reported to play a role in shaping the positioning of resident immune cells in the liver (29). However, CXCR3 expression in hepatic macrophages from *Smad4^fl/fl^* *Vav1^Cre^* mice was not affected (our unpublished observations), suggesting that the alteration of anatomical localization was not due to the lack of CXCR3 expression. In fact, monocytes constantly replenished the macrophage pool in the livers of *Alk1^fl/fl^* *Vav1^Cre^* mice and *Smad4^fl/fl^* *Vav1^Cre^* mice. These less-mature Mo-KCs may have contributed to a uniform distribution of KCs. Nevertheless, our study is the first to our knowledge to provide insights into how the tissue-specific functions of KCs are affected by factors that imprint their identity.

Deletion of *Alk1* and *Smad4* resulted in disruption of KC homeostasis, exhibited by loss of KCs over time and replacement by monocyte-derived macrophages. These immature monocyte-derived macrophages displayed reduced expression of VSIG4 and altered function of KCs. Finally, *Alk1/Smad4*-deficient mice showed an increased susceptibility to infection with gram-positive *L. monocytogenes*. In fact, mutations in *Alk1* result in hereditary hemorrhagic telangiectasia (HHT), which is a rare genetic disease characterized by recurrent epistaxis, cutaneous telangiectasia, and visceral arteriovenous malformations (AVMs) (40). It has been reported that patients with HHT are more susceptible to bacterial infection, especially gram-positive *Staphylococcus aureus* (41, 42). We also found that *Alk1^fl/fl^* *Vav1^Cre^* mice had a significant reduction in the capture of circulating *S. aureus* by KCs (Supplemental Figure 12). Thus, it is possible that the high incidence of infectious diseases observed in patients with HHT may be due to the impaired innate immune function of KCs seen in mice lacking ALK1. In addition, during liver injury or infection in mice, KC homeostasis was also disrupted, with loss of resident KCs and replenishment of monocyte-derived macrophages (43–45). More important, the alteration of KC homeostasis also occurs in human liver diseases, as the number of liver macrophages is significantly reduced in patients with liver fibrosis (46). It has been reported that patients with acute liver failure and advanced cirrhosis have a high risk of bacterial infections, and dysfunction of liver macrophages may play a role (46, 47). This suggests that therapeutic interventions aimed to prevent the loss of KCs and/or promote the maturation of newly arrived monocyte-derived macrophages might help reduce susceptibility to infection in these patients.

Recently, Bonnardel et al. and Sakai et al. proposed a 2-step model in which Notch signaling drives the initial commitment of BM monocytes to the KC lineage, and TGF-β family ligands, including TGF-β and BMP9, establish KC identity (6, 7). In addition, Sakai et al. documented an important role for Smad4 in maintaining KC identity (7). The 2 reports provided the background for understanding how engrafted circulating monocytes differentiated into KCs. In the present study, we precluded the roles of TGF-β, BMP2, and BMP6 and showed that, in addition to BMP9, BMP10 also has a critical role in maintaining KC identity. In addition, we also demonstrated that this signaling pathway is important for KCs to accomplish their strategic role in protecting the host from *L. monocytogenes* infection. Altogether, this study revealed how the liver microenvironment regulates KCs to maintain their phenotypes and form an effective defense against bacterial infection.

## Methods

### Mice.

*Clec4f^Cre/DTR^* mice were generated at the Nanjing BioMedical Research Institute of Nanjing University (NBRI) using CRISPR/Cas9-mediated genome editing on a C57/BL/6J background. *R26^tdTomato^* mice (stock no. 007914) and *UBC^CreERT2^* mice (stock no. 007001) were obtained from The Jackson Laboratory. *Tgfbr2^fl/fl^* mice and *Smad4^fl/fl^* mice were provided by Xiao Yang (Beijing Institute of Lifeomics). *Bmp9*-KO mice were provided by Se-Jin Lee (Johns Hopkins University, Baltimore, Maryland, USA). *Alk1^fl/fl^* mice were provided by Zhihong Xu (Fudan University, Shanghai, China). *Alk2^fl/fl^* mice were provided by Vesa Kaartinen (University of Michigan, Ann Arbor, Michigan, USA). *Alk3^fl/fl^* mice were provided by Yuji Mishina (University of Michigan). *Vav1^Cre^* mice were provided by Bing Liu (Fifth Medical Center of Chinese PLA General Hospital, Beijing, China). CD45.1/Ly5.1 mice were provided by Mingzhao Zhu (Institute of Biophysics, Chinese Academy of Sciences, Beijing, China). Mice and their littermates were used between 6 and 16 weeks of age unless otherwise specifically indicated. All mice were maintained at the specific pathogen–free (SPF) facilities of the Beijing Institute of Lifeomics.

### Cell suspension preparations, flow cytometry, and antibodies.

Cell suspensions were prepared as previously described (48). Briefly, CNS, spleen, lung, and kidney were cut into small pieces, incubated in collagenase type IV (MilliporeSigma) at 37°C for 30 minutes, and vigorously pipetted. The cell suspensions were filtered through a 70μm cell strainer to obtain a homogeneous cell suspension. CNS cell suspensions were further enriched by a Percoll gradient. The liver was perfused via the portal vein with approximately 20 mL HBSS, followed by perfusion with digestion buffer containing 0.05% collagenase type IV for 5 minutes. The digested livers were then excised and disrupted, and the cell suspension was passed through a 70μm cell strainer. Parenchymal cells were separated from nonparenchymal cells by centrifugation at 50*g* for 5 minutes. Liver cell suspensions were further enriched by iodixanol gradient (OptiPrep) as previously described (49).

For surface marker analysis, cell pellets were stained with the appropriate antibodies at 4°C for 20–30 minutes. For intracellular cytokine analysis, cells were stained with the Cytofix/Cytoperm kit according to the manufacturer’s instructions (eBioscience). To obtain hepatic mononuclear cells, mice were perfused via the portal vein with HBSS, and the livers were minced through a 70 μm cell strainer followed by lysis with RBC lysis buffer. The hepatic mononuclear cells were stimulated with a cell stimulation cocktail (eBioscience) for 6 hours, followed by intracellular IL-17A staining.

Flow cytometry was performed using an LSR II Fortessa (BD Biosciences). The acquired data were analyzed with FlowJo software (Tree Star). For cell sorting, a FACS Aria III (BD Biosciences) was used. The antibodies used are listed in Supplemental Table 2.

### In vitro culture of KCs.

Sorted KCs by FACS were seeded in 12-well plated in DMEM (HyClone) containing 10% FBS (Gibco, Thermo Fisher Scientific). After overnight, the culture medium was replaced by serum-free X-VIVO15 media (Lonza), supplemented with 20 ng/mL M-CSF in the presence or absence of 50 ng/mL rmBMP2 (Peprotech), rhBMP6 (Peprotech), rhBMP9 (Peprotech), or 50 ng/mL rmBMP10 (R&D Systems). The half media were changed every other day. On day 7, KCs were acquired, and RNA was extracted using an RNeasy Plus Mini Kit (QIAGEN).

### BM chimeras.

To establish total body irradiation, C57BL/6 (CD45.1) mice were lethally irradiated (10 Gy) by x-ray and transplanted with 1 × 10^7^ BM cells. The mice were analyzed 8 weeks after transplantation.

For establishment of partially shielded irradiation, livers of *Alk1^fl/fl^* *Clec4f^Cre^* mice (CD45.2) and *Smad4^fl/fl^* *Clec4f^Cre^* mice (CD45.2) or their WT controls (CD45.2) were protected by a lead cover and then irradiated (8 Gy) by x-ray. The irradiated mice were transplanted with 1 × 10^7^ BM cells from sex-matched WT mice (CD45.1). Four weeks after transplantation, the mice were analyzed.

### EdU staining.

Mice were administrated 0.5 mg EdU (Thermo Fisher Scientific) via i.p. injection. After 20 hours, KCs were obtained and EdU incorporation was measured by flow cytometry using the Click-iT EdU Alexa Fluor 647 Flow Cytometry Assay Kit (Thermo Fisher Scientific), according to the manufacturer’s instructions.

### qPCR.

Total RNA was isolated with RNeasy Plus Mini Kit (QIAGEN), and cDNA was synthesized with Prime Script RT Reagent Kit (Takara). qPCR was performed with a SYBR Green PCR kit in a CFX Connect Real-time PCR detection system (Bio-Rad). The specific qPCR primers used are listed in Supplemental Table 3.

### PCR analysis for Alk1 deletion.

Liver cells from *Alk1^fl/fl^ Clec4f^Cre^* mice were first isolated by F4/80 magnetic beads (Miltenyi Biotec) and then sorted by FACS. Genomic DNA was extracted using the QIAamp DNA Micro Kit (QIAGEN). PCR was performed as follows: 95°C for 3 minutes, 35 cycles at 95°C for 30 seconds, 62°C for 30 seconds, and 72°C for 45 seconds, followed by a 2-minute incubation at 72°C. PCR primers for floxed *Alk1* were as follows: forward, 5′-GCTTGCATGCTTGGCTCTAC-3′; reverse, 5′-GGGAGGAGCCATGTTCTCAG-3′. PCR primers for *Alk1* deletion were as follows: forward, 5′-GTGGCTGGAGAGGAACAGTAGTCC-3′; reverse, 5′-TGGAGACCTGCTCTGAGATGTCTG-3′. (See complete unedited blots int the supplemental material.)

### Bacterial burden.

Mice were injected with *L. monocytogenes* (strain 10403s) via the tail vein. The livers and spleens at the indicated post-infection time points were removed and homogenized. Serial dilutions of cell suspensions in PBS containing 1% Triton X-100 were seeded on brain heart infusion (BHI) agar plates. After overnight incubation at 37°C, CFU were counted.

### scRNA-Seq.

Single Tim4^+^CD64^+^F4/80^+^Ly6C^–^CD45^+^ cells were sorted from the livers of *Alk1^fl/fl^* *Clec4f^Cre^* and their *Alk1^fl/fl^* littermate controls. FACS-purified cells were washed with 0.04% BSA DPBS 3 times and resuscitated to a concentration of 700–1200 cells/μL (viability ≥85%). Individual cells were then loaded onto a 10x Genomics Chromium controller to generate single-cell Gel Bead-in-EMulsion (GEMs). Single-cell sequencing libraries were prepared using 10x Genomics Chromium Single Cell 3′ Reagent kits (v3 Chemistry) and sequenced on the NovaSeq 6000 platform (Illumina). Raw reads were demultiplexed and aligned to the mouse transcriptome (mm10) using the 10x Genomics Cell Ranger pipeline (version 3.1.0). All downstream single-cell analyses were performed with R package Seurat (version 3.2.2). Additional low-quality (expressed genes <500, unique molecular identifier [UMI] counts <2000, mitochondrial genes <10% or ribosomal genes <14%) and contaminating cells (lymphocytes) were removed from the analysis.

### Data availability.

The raw RNA-Seq data generated from this study are available in the NCBI’s Sequence Read Archive (SRA) under accession code PRJNA705814.

### IVM of liver and L. monocytogenes capture.

A multichannel confocal microscope was used to image mouse liver as previously described (50). Briefly, mice were anesthetized (2.5% avertin, 20 mL/kg, i.p., MilliporeSigma). The tail vein was cannulated to administer fluorescent dyes and labeled bacteria. The abdominal cavity was exposed by removing the skin and muscles. The mouse was placed on a heated stage (37°C), and the largest lobe of the liver was positioned onto a coverslip; a small piece of sterile laboratory wipes was moisturized with saline and placed over the liver to keep it moist and stable. For the visualization of liver macrophages, platelets, and neutrophils, 2 μg anti-F4/80 (BM8) or 2.5 μg anti-Ly6G (1A8) (BioLegend) was administered i.v. Images were acquired using an inverted Olympus FV3000 confocal microscope with a 20×/0.75 UPLANSAPO objective lens. Laser excitation wavelengths of 488 nm, 561 nm, and 647 nm and a high-sensitivity spectral detector with a GaAsP photomultiplier tube (PMT) or a spectral detector with multialkali PMTs were used for fluorescence detection. For bacteria-catching experiments, bacteria were resuspended in PBS and labeled by incubation with CFSE (100 μM, Invitrogen, Thermo Fisher Scientific) for 30 minutes. Acquisition of images was initiated, labeled bacteria (4 × 10^7^ CFU) were injected via the tail vein immediately, and images were acquired every 5 seconds for 25 minutes. Functions of colocalization and spot in the Imaris software were used to identify and quantify captured bacteria within KCs per field of view at every time point.

For the visualization of cell interaction, acquisition of images was initiated, 5 × 10^8^ *L. monocytogenes* was injected via the tail vein, and images were acquired every 10 seconds for 50 minutes. A stitched image of 4 × 4 fields of view was acquired 2 hours after infection to visualize neutrophil recruitment. Functions of colocalization and surface in the Imaris software (Bitplane) were used to measure voxel overlap between 2 cell surfaces and to identify interactions between KCs and platelets or neutrophils.

### Immunofluorescence staining.

Livers were perfused with 0.5% heparin (ADAMAS-BETA) in 20 mL cold PBS and fixed in 4% paraformaldehyde (PFA) for 12 hours, followed by dehydration in 15% and 30% sucrose before embedment in OCT compound (Sakura Finetek). Sections (50 μm thick) were cut on a CM1950 cryostat (Leica) and adhered to Superfrost Plus slides. Frozen sections were blocked for 2 hours with 1% BSA, 0.3 M glycine, and 10% donkey or goat serum after permeabilization in PBS with 0.2% Triton X-100 for 30 minutes at room temperature, followed by staining with antibodies diluted in PBS with 0.2% Tween-20 for 12 hours at 4°C and with secondary antibodies and Hoechst for 2 hours at room temperature. The following antibodies were used: anti-CD31 (390, BD Bioscience), anti-F4/80 (BM8, BioLegend), anti–E-cadherin (DECMA-1, BioLegend), anti-desmin (polyclone, Abcam), anti–glutamine synthetase (polyclone, Abcam), anti-VSIG4 (NLA14, Invitrogen, Thermo Fisher Scientific), and anti-Clec4F (polyclone, R&D Systems). Fluorescence-conjugated secondary antibodies were purchased from Jackson ImmunoResearch Laboratories. Sections were mounted with Fluoromount G (Yeasen Biotechnology), and images were acquired on a FV3000 confocal microscope with a 20×/0.75 UPLANSAPO or 60×/1.30 Sil UPLANSAPO objective lens. Laser excitation wavelengths of 405 nm, 488 nm, 561 nm, and 647 nm and HyD or PMT detectors were used for fluorescence detection.

### Image processing and analysis.

All videos and images were processed using Fiji (NIH) or Imaris (Bitplane) software. KC numbers, cell surface area, and cell volume were quantified using the surface function in Imaris. The surface was created for F4/80, the threshold was determined by a background subtraction algorithm, and the fluorescence intensity of Clec4F and VSIG4 for every individual KC was calculated. The positions of KCs were obtained and the distance to the center of each central vein (CV) and portal vein (PV) was calculated in Microsoft Excel.

### Statistics.

Data are presented as the mean ± SEM. All statistical analyses were performed using GraphPad Prism (GraphPad Software). Statistical significance was assessed by an unpaired, 2-tailed Student’s *t* test, a 1-way ANOVA with Tukey’s multiple-comparison test, or a 2-way ANOVA with Sidak’s multiple-comparison test where appropriate. Survival analysis was assessed with the Mantel-Cox test. Each symbol represents an individual mouse.

### Study approval.

All experimental procedures using mice were approved by the IACUC of the Beijing Institute of Lifeomics.

## Author contributions

DZ conceived the study, acquired funding, designed and performed the experiments, analyzed and curated the majority of the data, interpreted results, and wrote the manuscript. FY performed the experiments, analyzed the data, edited the manuscript, and performed imaging. YW performed the experiments, analyzed the data, and performed imaging. SL performed the experiments and imaging analysis. YL performed bioinformatics analysis. F Hou provided technical and material support. WY, DL, YT, and QL provided technical support. LT conceived the study, edited the manuscript, acquired funding, and supervised the study. JW edited the manuscript, acquired funding, and supervised the study. F He supervised the study. The order of the co–first authors, DZ, FY, YW, and SL, was determined on the basis of their contributions to this project.

## Supplementary Material

Supplemental data

Supplemental video 1

Supplemental video 2

Supplemental video 3

## Version 1 12/07/2021

In-Press Preview

## Version 2 02/01/2022

Electronic publication
