# Supplementary material for: ALK1 signaling is required for the homeostasis of Kupffer cells and prevention of bacterial infection
Source: J Clin Invest. 2022 Feb 1;132(3):e150489. doi: 10.1172/JCI150489 (PMC8803331; doi:10.1172/JCI150489)

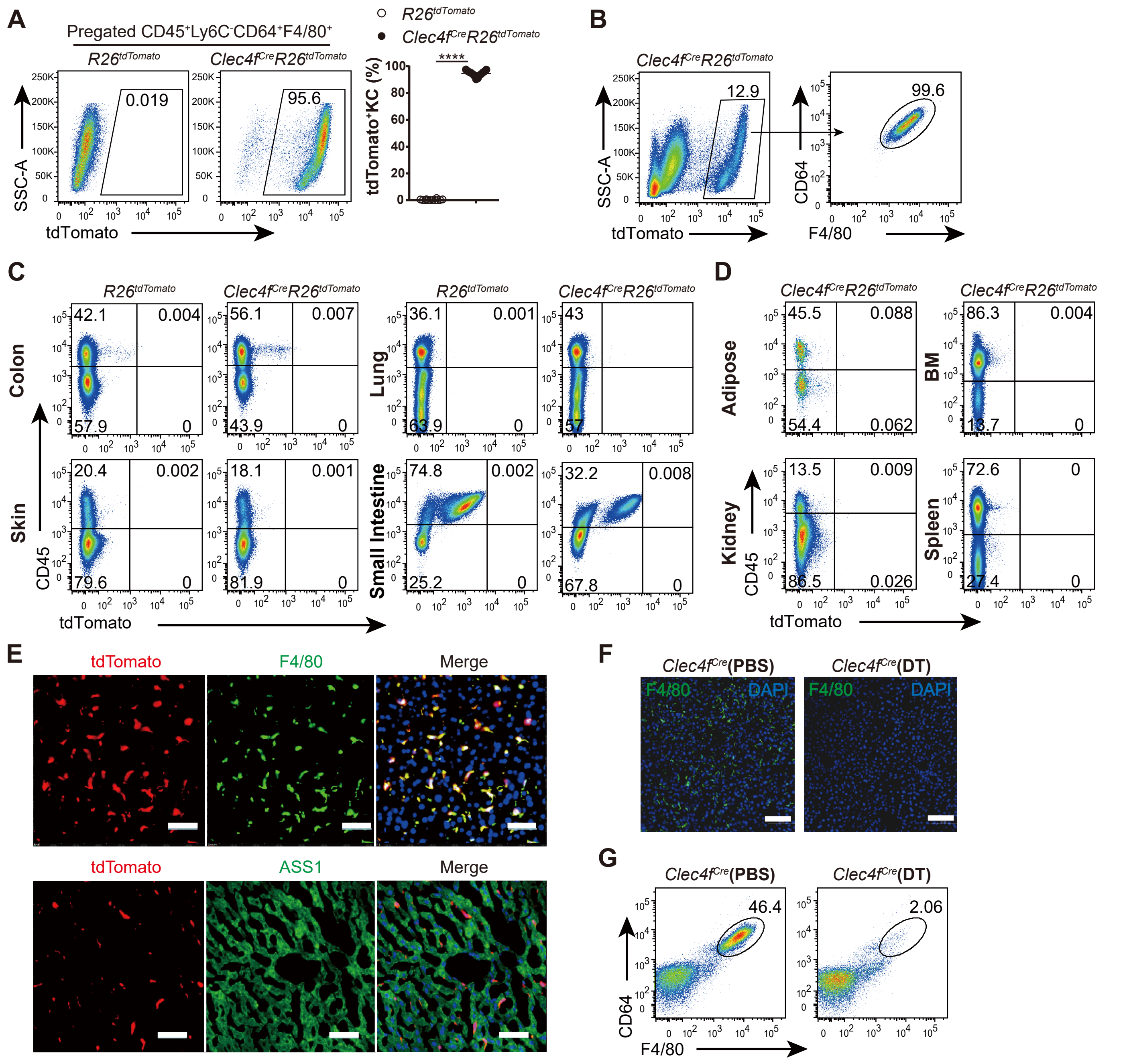

Supplemental Figure 1. KCs are specificially targeted in $Clec4f^{Cre}R26^{tdTomato}$ mice.

(A) Representative flow cytometry data (left) and percentage (right) of reporter gene expression in KCs (pre-gated $CD45^{+}Ly6C^{-}CD64^{+}F4/80^{+}$) from $Clec4f^{Cre}R26^{tdtomato}$ and $R26^{tdtomato}$ mice (n = 12-13 per group). Gating strategy for KCs was shown in Figure. S2A. The results represent mean ± SEM. ****p < 0.0001, calculated by two-tailed Student's t-test.

(B) Flow cytometry analysis of F4/80 and CD64 in tdTomato-positive cells from $Clec4f^{Cre}R26^{tdtomato}$ mice, Data are representative of at least three independent experiments.

(C and D) Reporter gene expression in other tissues and BM from $Clec4f^{Cre}R26^{tdtomato}$ and $R26^{tdtomato}$ mice. Data are representative of two independent experiments.

(E) Immunostaining of liver of $Clec4f^{Cre}R26^{tdtomato}$ mice, showing tdTomato (red) and staining with DAPI (blue), F4/80 (KCs, green) and ASS1 (hepatocyte, green) (original magnification ×40; scale bar represents 50 μm).

(F) Expression of F4/80 and DAPI by confocal microscopy 24h post administration of DT or PBS to $Clec4f^{Cre}$ mice (scale bar represents 100 μm). The experiment was repeated two times.

(G) KCs ($CD45^{+}Ly6C^{-}CD11b^{+}F4/80^{+}$) were examined by flow cytometry 24h post administration of DT or PBS to $Clec4f^{Cre}$ mice. Data are representative of at least three independent experiments.

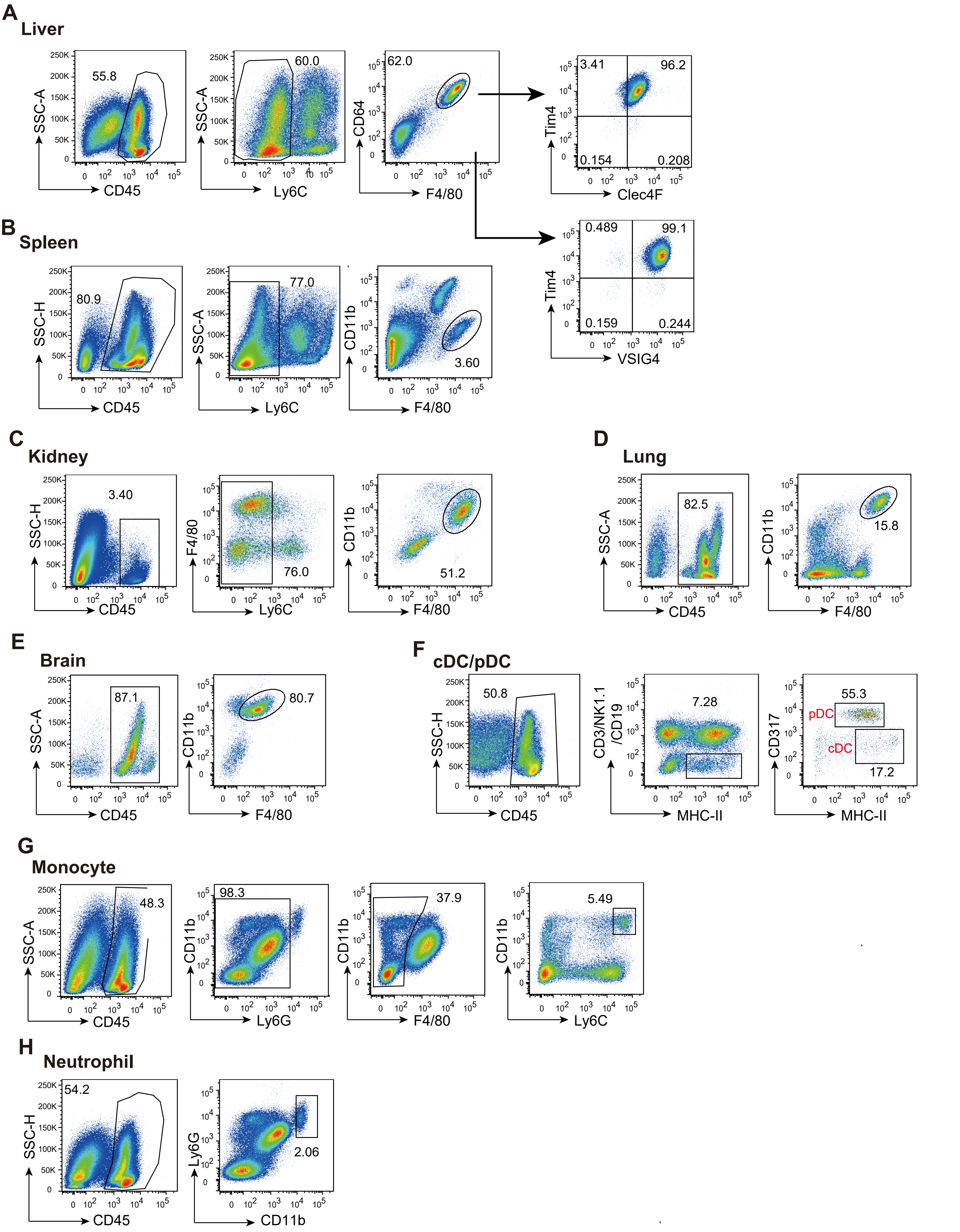

Supplemental Figure 2. Gating strategies.

(A-E) Gating strategies for KC in the liver (A), red pulp macrophage in the spleen (B), macrophage in the kidney (C), alveolar macrophage in the lung (D), macrophage in the brain (E), cDC and pDC in the liver (F), monocyte (G) and neutrophil (H) in the liver. Cells were pre-gated on singlets.

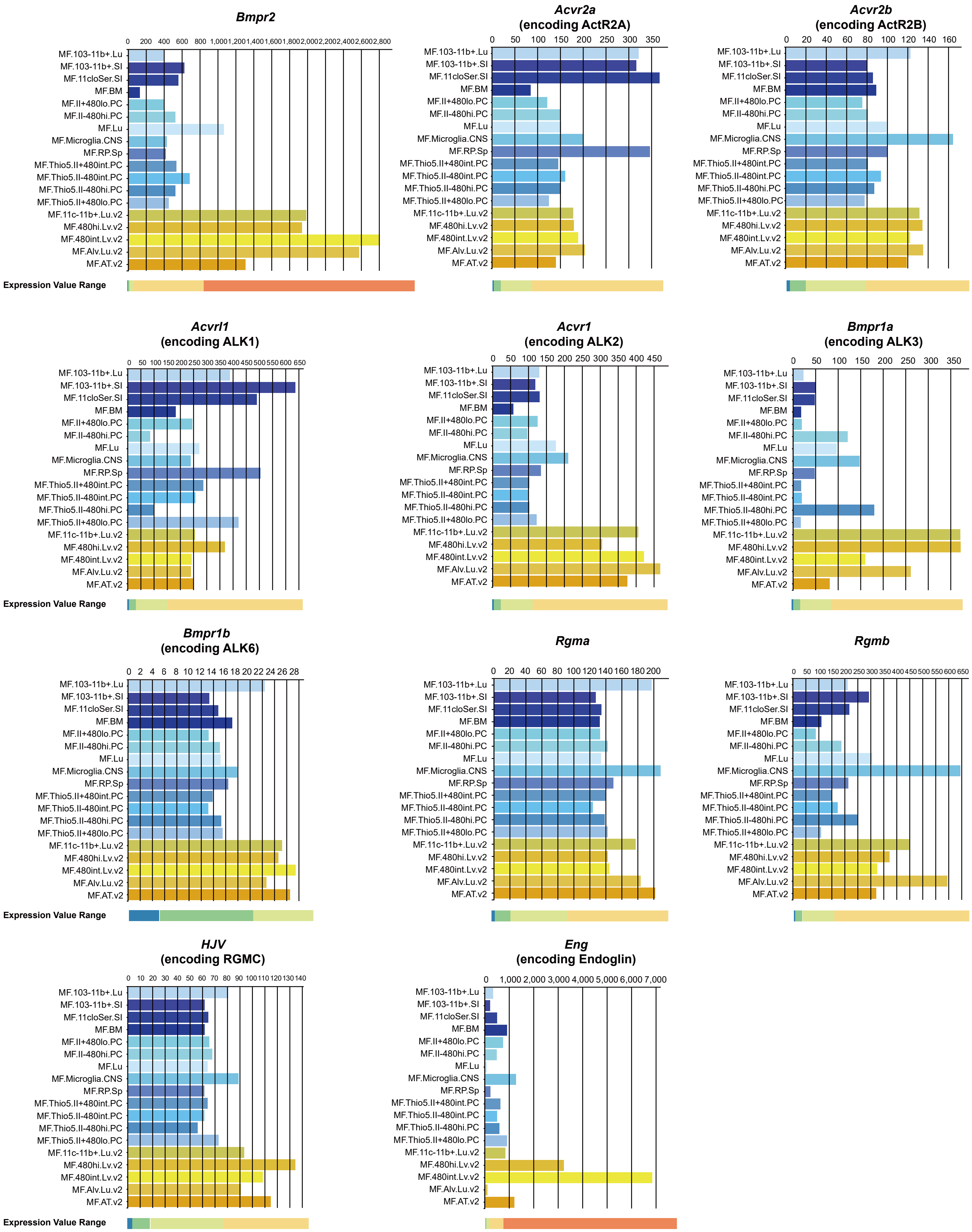

Supplemental Figure 3. Graphs from Immgen Consortium platform showing indicated genes expression of macrophages located in different organs.

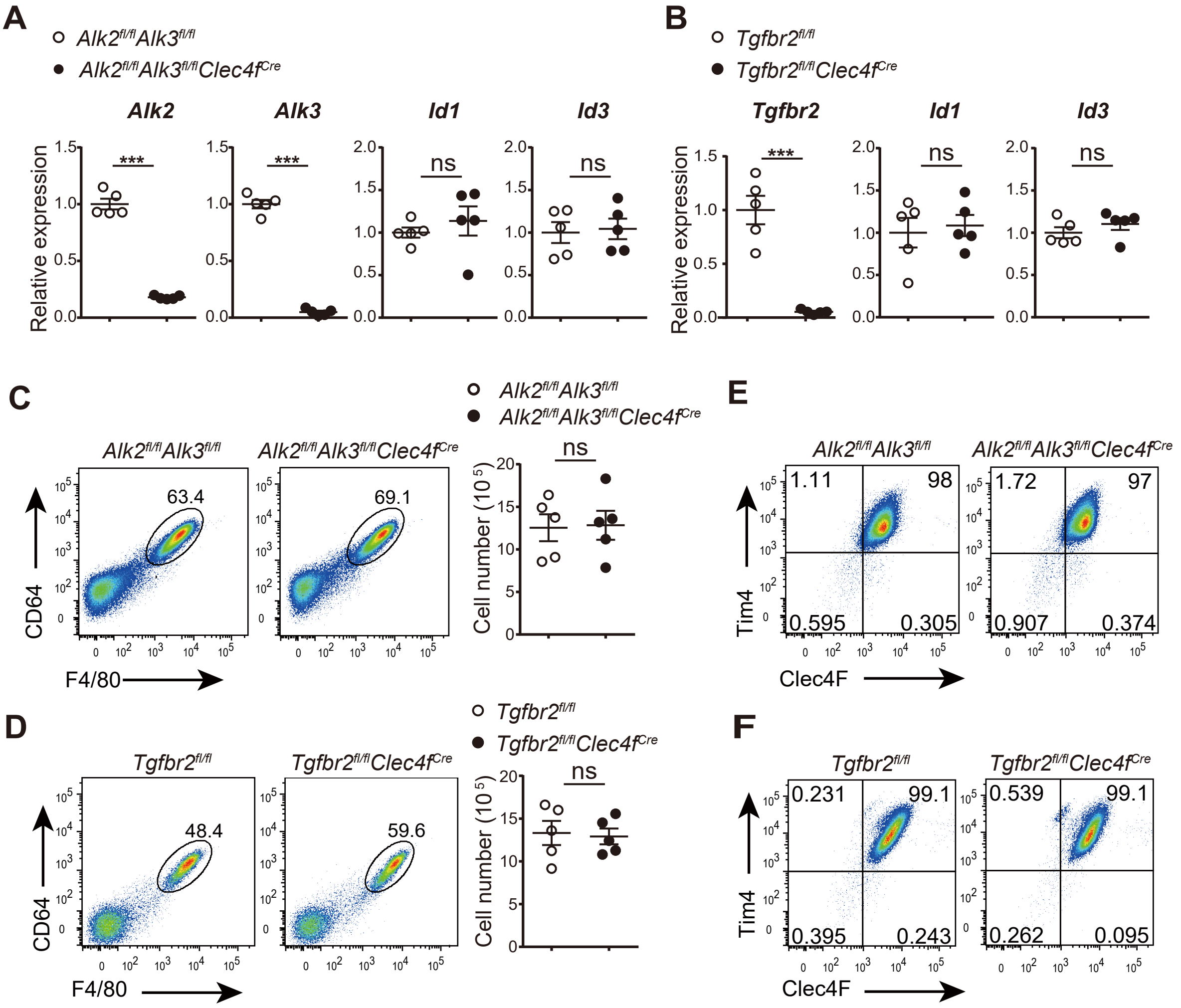

Supplemental Figure 4. Loss of ALK2, ALK3 and TGFβR2 does not affect KC surface phenotype.

(A-B) qPCR analysis of *Alk2*, *Alk3*, *Tgfbr2*, *Id1* and *Id3* expression in sorted KCs (CD45^{+}Ly6C^{-}CD64^{+}F4/80^{+}) from indicated mice (*n* = 5 per group). Gating strategy for KCs was shown in Supplemental Figure 2A.

(C and D) Representative flow cytometry data (left) and total cell number (right) of KCs (pregated on CD45^{+}Ly6C^{-}) in *Alk2$^{fl/fl}$ Alk3$^{fl/fl}$Clec4f^{Cre}* mice (C), *Tgfbr2$^{fl/fl}$Clec4f^{Cre} mice* (D) and their controls (*n* = 5 per group).

(E and F) Expression of Clec4F and Tim4 in KCs from *Alk2$^{fl/fl}$Alk3$^{fl/fl}$ Clec4f^{Cre}* mice (E), *Tgfbr2$^{fl/fl}$Clec4f^{Cre}* mice (F) and their controls. Data are representative of at least three independent experiments. The results represent mean ± SEM. ***P < 0.001, calculated by two-tailed Student's t-test (A-D).

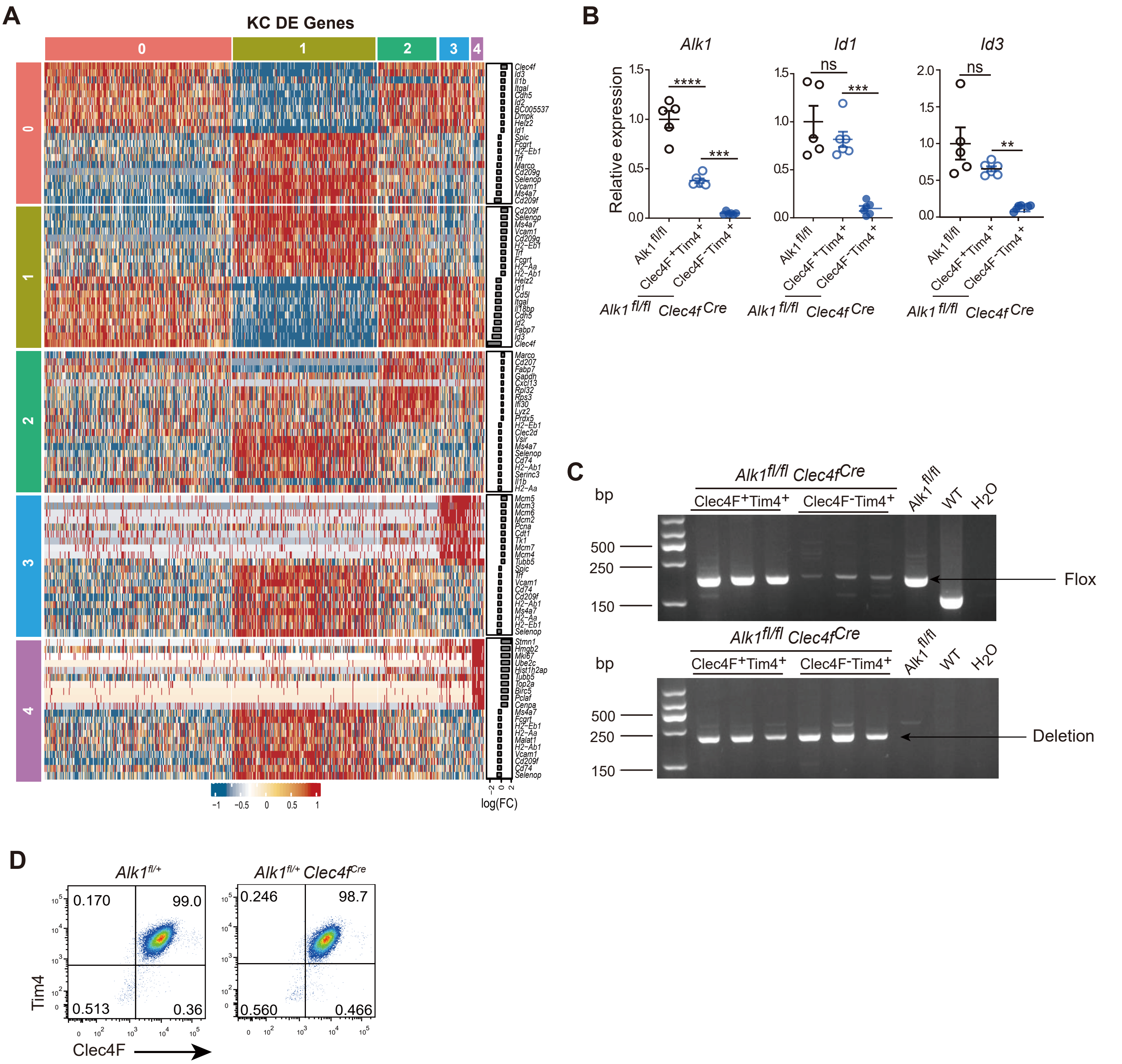

Supplemental Figure 5. ***Alk1*** **is efficiently deleted in Clec4F⁻Tim4⁺ KCs from** ***Alk1*** $^{fl/fl}$ ***Clec4f*** ^{Cre} **mice.**
(A) Heatmaps showing top DE genes (10 upregulated, 10 downregulated) per cluster of KCs based on LogFC.
(B) qPCR analysis of *Alk1*, *Id1* and *Id3* expression in sorted Clec4F⁺Tim4⁺ and Clec4F⁻Tim4⁺ KCs from *Alk1*$^{fl/fl}$-*Clec4f*^{Cre} mice relative to sorted KCs from *Alk1*$^{fl/fl}$ controls (*n* = 5-6 per group).
(C) Genomic PCR analysis for *Alk1* flox and *Alk1* deletion band in sorted Clec4F⁺Tim4⁺ and Clec4F⁻Tim4⁺ KCs from *Alk1*$^{fl/fl}$*Clec4f*^{Cre} mice compared with *Alk1*$^{fl/fl}$ and WT controls.
(D) Flow cytometry of Clec4F and Tim4 of KCs (pregated on CD45⁺Ly6C⁻CD64⁺F4/80⁺) in *Alk1*$^{fl/+}$*Clec4f*^{Cre} mice and *Alk1*$^{fl/+}$ controls. Data are representative of at least three independent experiments. The results represent mean ± SEM. ****P <0.0001, ***P <0.001, **P <0.01, calculated by one-way ANOVA (B).

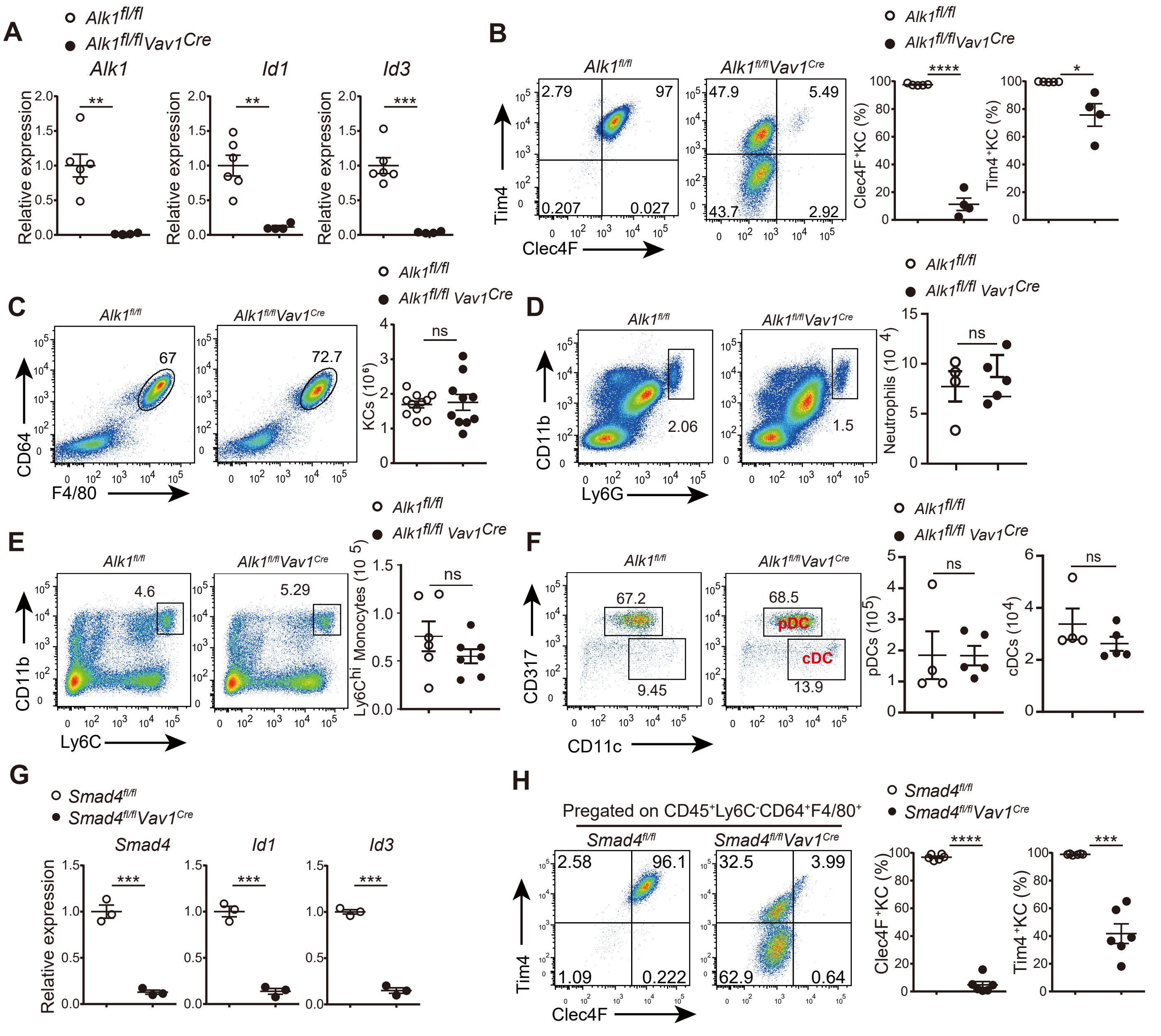

Supplemental Figure 6. *Alk1* and *Smad4* are efficiently targeted in KCs from *Alk1$^{fl/fl}$Vav1^{Cre}* and *Smad4$^{fl/fl}$Vav1^{Cre}* mice, respectively.

(A) qPCR analysis of *Alk1*, *Id1* and *Id3* expression in sorted KCs (CD45^{+}Ly6C^{-}CD64^{+}F4/80^{+}) from *Alk1$^{fl/fl}$Vav1^{Cre}* mice and *Alk1$^{fl/fl}$* controls (n = 4-6 per group).

(B) Representative flow cytometry data (left) of Clec4F and Tim4 of KCs (pregated on CD45^{+}Ly6C^{-}CD64^{+}F4/80^{+}) and percentage (right) of Clec4F^{+} and Tim4^{+} KCs in *Alk1$^{fl/fl}$Vav1^{Cre}* mice and *Alk1$^{fl/fl}$* controls (n = 4 per group).

(C-F) Representative flow cytometry data (left) and total cell numbers (right) of KCs (C, pregated on CD45^{+}Ly6C^{-}, n = 10-11 per group), neutrophils (D, pregated on CD45^{+}, n = 4-5 per group), monocytes (E, pregated on CD45^{+}Ly6G^{-}F4/80^{-}, n = 6-7 per group), pDC and cDC (F, pregated on CD45^{+}CD3^{-}CD19^{-}NK1.1^{-}MHCII^{+}, n = 4-5 per group) in the liver of *Alk1$^{fl/fl}$Vav1^{Cre}* mice and *Alk1$^{fl/fl}$* controls. Each symbol represents an individual mouse. Gating strategies for KC, neutrophil, monocyte, pDC and cDC were shown in Supplemental Figure 2.

(G) qPCR analysis of *Smad4*, *Id1* and *Id3* expression in sorted KCs (CD45^{+}Ly6C^{-}CD64^{+}F4/80^{+}) from *Smad4$^{fl/fl}$Vav1^{Cre}* mice and *Smad4$^{fl/fl}$* controls (n = 3 per group).

(H) Representative flow cytometry data (left) of Clec4F and Tim4 of KCs (pregated on CD45^{+}Ly6C^{-}CD64^{+}F4/80^{+}) and percentage (right) of Clec4F^{+} and Tim4^{+} KCs in *Smad4$^{fl/fl}$Vav1^{Cre}* mice and *Smad4$^{fl/fl}$* controls (n = 6 per group). The results represent mean ± SEM. ****P <0.0001, ***P <0.001, **P <0.01, *P <0.05, calculated by two-tailed Student's t-test (A-H).

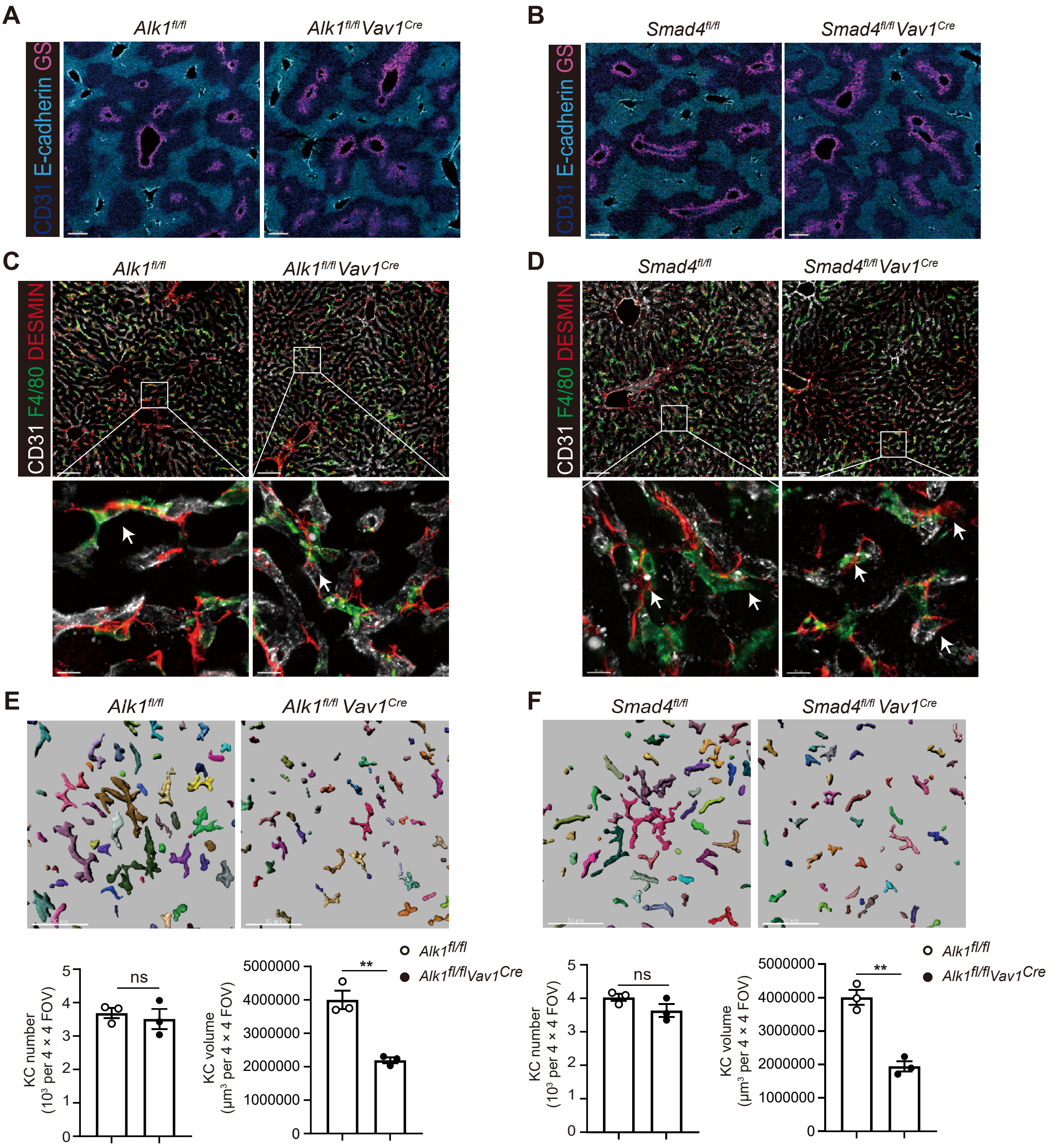

Supplemental Figure 7. ***$Alk1^{fl/fl}Vav1^{Cre}$* and *$Smad4^{fl/fl}Vav1^{Cre}$* mice have normal liver architecture.**

(A and B) Representative immunofluorescence image of liver sections stained with anti-E-cadherin (cyan), anti-Glutamine Synthetase (magenta) and anti-CD31 (blue) showing the liver metabolic zone distribution of *$Alk1^{fl/fl}Vav1^{Cre}$* mice (I), *$Smad4^{fl/fl}Vav1^{Cre}$* mice (J) and their littermate controls. Scale bar, 200 µm. Data are representative of three independent experiments.

(C and D) Representative immunofluorescence image (top) and zoom-in (bottom) of liver sections stained with anti-F4/80 (green), anti-Desmin (red) and anti-CD31 (white) showing the interaction between KCs, stellate cells and vascular endothelial cells (arrows) in *$Alk1^{fl/fl}Vav1^{Cre}$* mice (K), *$Smad4^{fl/fl}Vav1^{Cre}$* mice (L) and their littermate controls. Scale bar, 200 µm (top) and 10 µm (bottom). Data are representative of three independent experiments.

(E and F) Representative 3D reconstruction (top) and quantification data of KC number and total KC volume (bottom) showing the morphology of KCs from the livers of *$Alk1^{fl/fl}Vav1^{Cre}$* mice (E), *$Smad4^{fl/fl}Vav1^{Cre}$* mice (F) and their littermate controls (*n* = 3 per group). Non-specific coloring to demonstrate individual cells. Scale bar, 80 µm. The results represent mean ± SEM. **P <0.01, calculated by two-tailed Student's t-test (E, F).

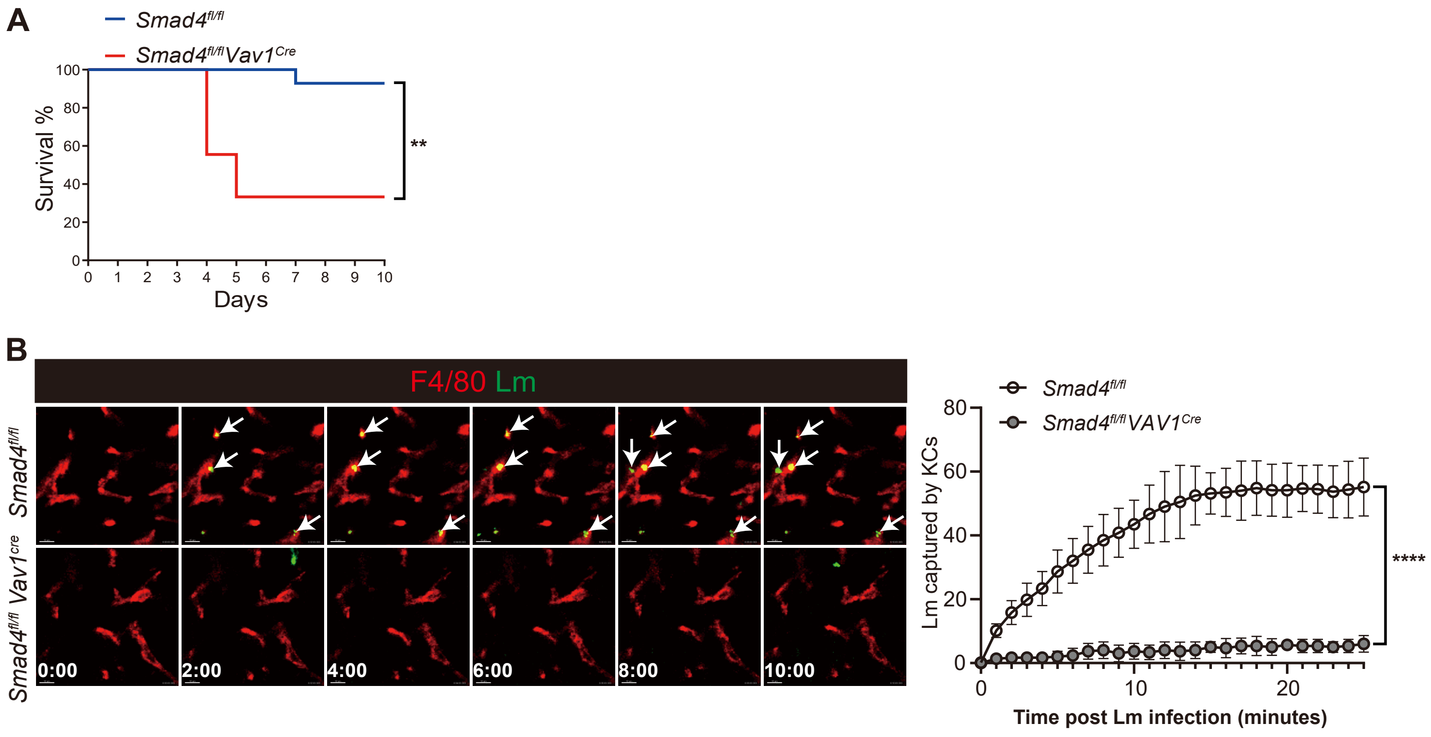

Supplemental Figure 8. ***Smad4*$^{fl/fl}$*Vav1*^{Cre} mice exhibit increased susceptibility to Lm infection.**

(A) Survival of *Smad4*$^{fl/fl}$*Vav1*^{Cre} mice (n = 9) and their littermate controls (n = 14) infected with 5×10^5 CFU Lm was monitored every day.

(B) Representative IVM images (left) and quantification (right) showing KCs (anti-F4/80, red) capturing circulating Lm (CFSE, green) within 25 minutes after infection in *Smad4*$^{fl/fl}$*Vav1*^{Cre} mice (n = 3) and their littermate controls (n = 6). Bacteria captured by Kupffer cells are highlighted (arrows). Scale bar, 10 µm. The results represent mean ± SEM. ****P <0.0001, **P <0.01, calculated by Mantel-Cox test (A), two-way ANOVA (B).

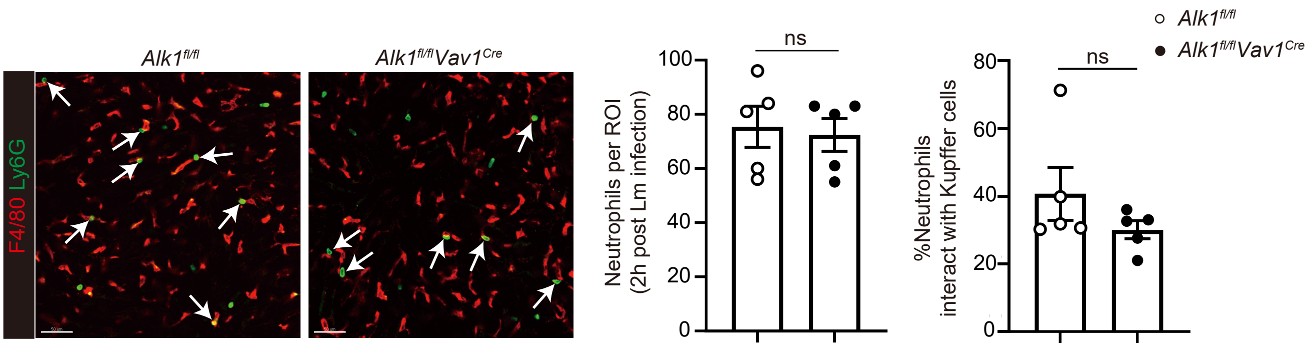

Supplemental Figure 9. Loss of ALK1 does not affect the recruitment of neutrophils and their interaction with KCs.

Representative IVM (intravital microscopy) images (left) showing interaction between KCs (anti-F4/80, red) and neutrophils (anti-Ly6G, green, arrows) in *Alk1$^{fl/fl}$* mice and *Alk1$^{fl/fl}$Vav1^{Cre}* mice 2 hours after Lm infection. Number of neutrophils (middle) in each region of interest (ROI) and the percentage of their interaction with KCs (right) were measured. Data are pooled from *n*=5 mice. Scale bar, 50μm. The results represent mean ± SEM. calculated by two-tailed Student’s t-test.

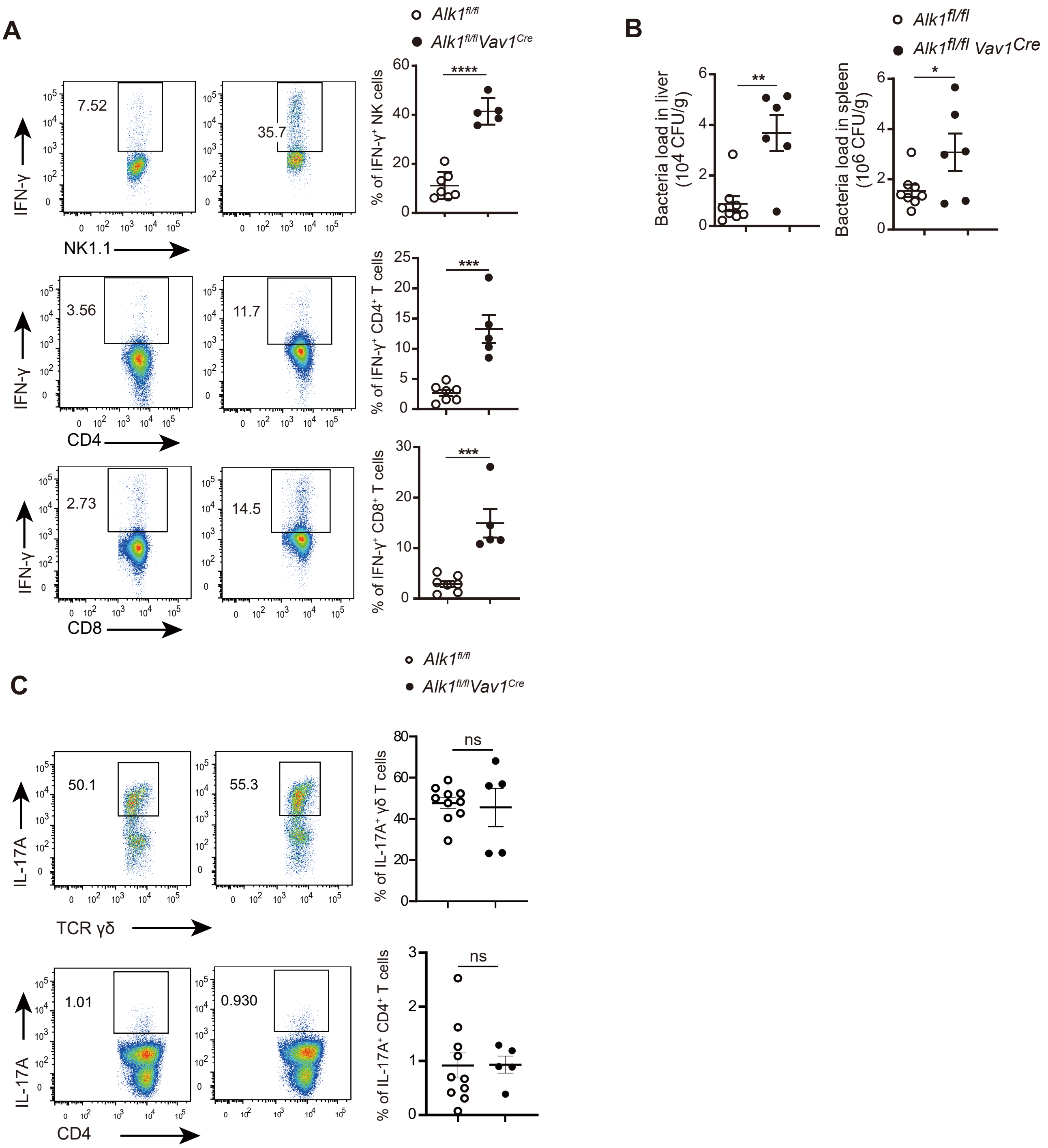

Supplemental Figure 10. Production of IFN-γ and IL-17A by immune cells in *Alk1$^{fl/fl}$Vav1^{Cre}* mice infected with Lm.

(A) Representative flow cytometry data of intracellular staining of IFN-γ in spleen NK cells (pregated on CD45$^+$CD3$^-$), CD4$^+$ T cells (pregated on CD45$^+$CD3$^+$) and CD8$^+$ T cells (pregated on CD45$^+$CD3$^+$) from *Alk1$^{fl/fl}$-Vav1^{Cre}* mice and *Alk1$^{fl/fl}$* controls 24h after Lm infection (1×10^5 CFU, *n* = 5-7 per group).

(B) Bacteria load in livers and spleens from *Alk1$^{fl/fl}$Vav1^{Cre}* mice and *Alk1$^{fl/fl}$* controls 24h after Lm infection (1×10^5 CFU, *n* = 6-8 per group).

(C) Representative flow cytometry data of intracellular staining of IL-17A in liver γδT cells (pregated on CD3$^+$NK1.1$^-$), CD4$^+$ T cells (pregated on CD3$^+$NK1.1$^-$) from *Alk1$^{fl/fl}$Vav1^{Cre}* mice and *Alk1$^{fl/fl}$* controls 5 days after Lm infection (1×10^5 CFU, *n* = 5-10 per group). The results represent mean ± SEM. ****P <0.0001, ***P <0.001, **P <0.01, *P <0.05, calculated by two-tailed Student's t-test (A, B and C).

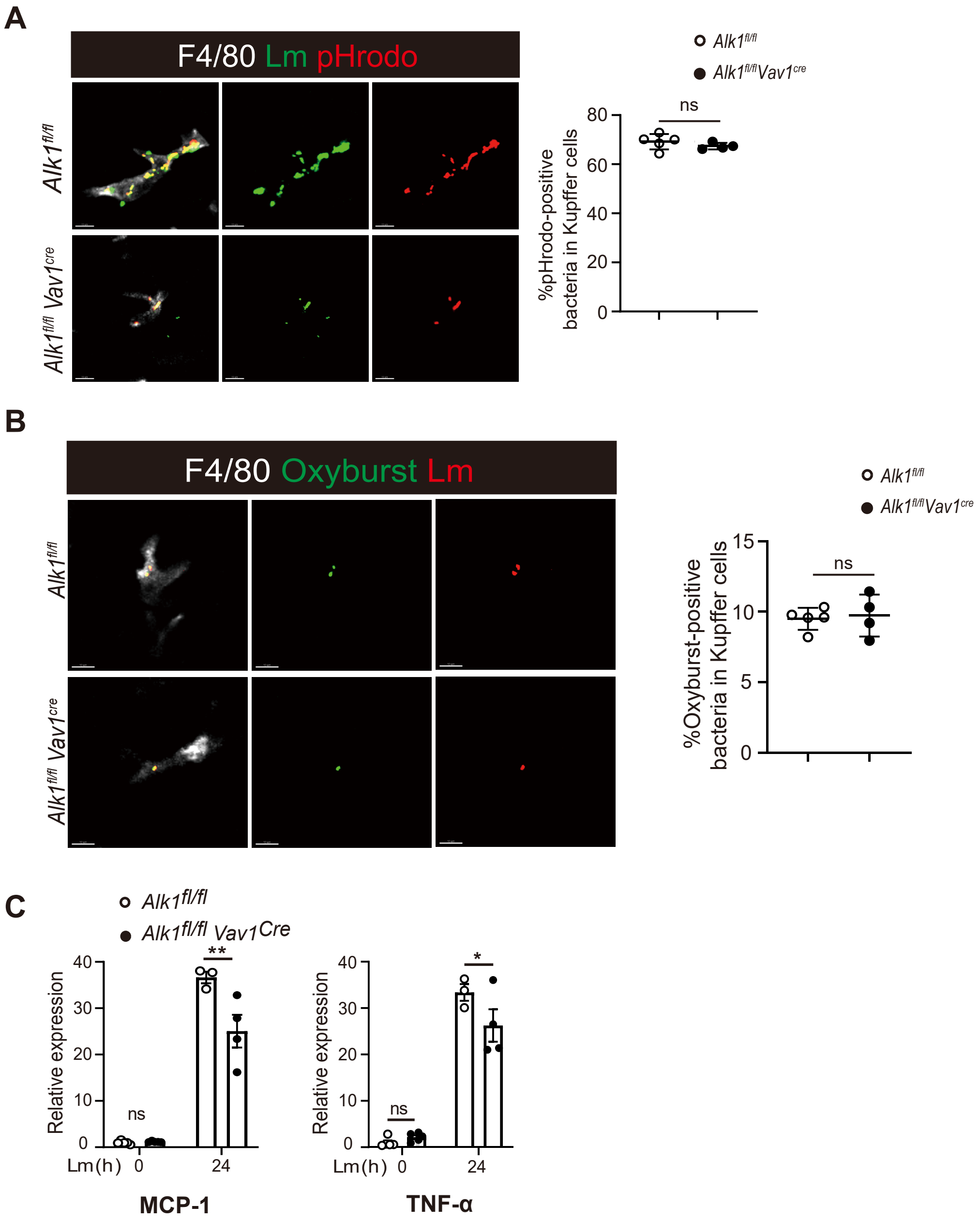

Supplemental Figure 11. Phagosome maturation, ROS production and inflammatory cytokine expression in KCs of *Alk1$^{fl/fl}$Vav1^{Cre}* mice and *Alk1$^{fl/fl}$* controls.

(A) Representative IVM images (top) assessing lysosome acidification (pHrodoRed, red) within KCs (anti-F4/80, white) in *Alk1$^{fl/fl}$* mice and *Alk1$^{fl/fl}$Vav1^{Cre}* mice (*n* = 4-5 per group) 1 hour after Lm (CFSE, green) infection and percentage of acidified (pHrodoRed-positive) bacteria (bottom). Data are pooled from two independent experiments. Scale bar, 10 μm.

(B) Representative IVM images (top) assessing ROS production (OxyBURST, green) within KCs (anti-F4/80, white) in *Alk1$^{fl/fl}$* mice and *Alk1$^{fl/fl}$Vav1^{Cre}* mice (*n* = 4-5 per group) 1 hour after Lm (Syto 60, red) infection and percentage of ROS-positive bacteria (bottom). Data are pooled from two independent experiments. Scale bar, 10 μm.

(C) qPCR analysis of *Mcp-1* and *Tnf-α* expression in sorted KCs ($CD45^+Ly6C^-CD64^+F4/80^+$) from *Alk1$^{fl/fl}$Vav1^{Cre}* mice and *Alk1$^{fl/fl}$* controls (*n* = 3-5 per group) with or without 1×10^5 CFU Lm infection. Scale bar, 10 μm. The results represent mean ± SEM. **P <0.01, *P <0.05, were calculated by two-tailed Student's t-test (A,B) and two-way ANOVA(C).

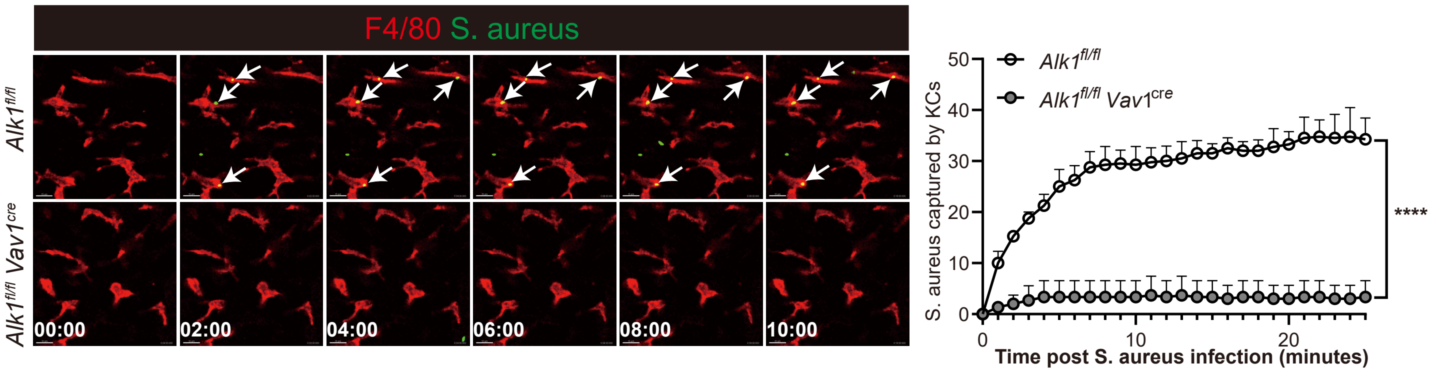

Supplemental Figure 12. ***Alk1***$^{fl/fl}$***Vav1***^{Cre} **mice mice show a significant reduction in the capture of circulating S.aureus by KCs.**

Representative IVM images (left) and quantification (right) showing KCs (anti-F4/80, red) capturing circulating *S.aureus* within 25 minutes after infection in *Alk1*$^{fl/fl}$*Vav1*^{Cre} mice and their littermate controls (n = 3 per group). Bacteria captured by Kupffer cells are highlighted (arrows). Scale bar, 10 μm. The results represent mean ± SEM. ****P < 0.0001, calculated by two-way ANOVA.

Supplemental Movie Legends

Supplemental Movie 1.

Circulating bacterial capture by KCs in *Alk1$^{fl/fl}$* and *Alk1$^{fl/fl}$Vav1^{Cre}* mice.

Confocal intravital liver microscopy of *Alk1$^{fl/fl}$* mice (left) and *Alk1$^{fl/fl}$Vav1^{Cre}* mice (right) showing capture of circulating Lm (CFSE, green) by KCs (PE-F4/80, red) within 25 minutes after i.v. infection with 4×10^7 CFU. Scale bar, 50μm.

Supplemental Movie 2.

Circulating bacterial capture by KCs in *Alk1$^{fl/fl}$* and *Alk1$^{fl/fl}$Clec4f$^{Cre/Cre}$* mice.

Confocal intravital liver microscopy of *Alk1$^{fl/fl}$* mice (left) and *Alk1$^{fl/fl}$Clec4f$^{Cre/Cre}$* mice (right) showing capture of circulating Lm (CFSE, green) by KCs (PE-F4/80, red) within 25 minutes after i.v. infection with 4×10^7 CFU. Scale bar, 50μm.

Supplemental Movie 3.

Circulating bacterial capture by KCs in *Smad4$^{fl/fl}$* mice and *Smad4$^{fl/fl}$Vav1^{Cre}* mice.

Confocal intravital liver microscopy of *Smad4$^{fl/fl}$* mice (left) and *Smad4$^{fl/fl}$Vav1^{Cre}* mice (right) showing capture of circulating Lm (CFSE, green) by KCs (PE-F4/80, red) within 25 minutes after i.v. infection with 4×10^7 CFU. Scale bar, 50μm.

Supplemental Table 1. DE Genes across clusters from Tim4+ KCs sorted from *Alk1*$^{fl/fl}$*Clec4f*^{Cre} and *Alk1*$^{fl/fl}$ mice (Red = Up, Blue = Down, compared with all other clusters).

Cluster 0	Cluster 1	Cluster 2	Cluster 3	Cluster 4
Clec4f	Cd209f	Marco	Mcm5	Stmn1
Id3	Selenop	Cd207	Mcm3	Hmgb2
Il1b	Ms4a7	Fabp7	Mcm6	Mki67
Itgal	Vcam1	Gapdh	Mcm2	Ube2c
Cdh5	Cd209g	Cxcl13	Pcna	Hist1h2ap
Id2	H2-Eb1	Rpl32	Cdt1	Tubb5
BC005537	Trf	Rps3	Tk1	Top2a
Dmpk	Fcgrt	Ifi30	Mcm7	Birc5
Helz2	H2-Aa	Lyz2	Mcm4	Pclaf
Id1	H2-Ab1	Prdx5	Tubb5	Cenpa
Fabp7	Spic	Cela1	Tuba1b	H2afz
Il18bp	Vsir	Rps2	Lig1	Ccnb2
Gpx1	Serinc3	Txn1	Rrm1	Kif23
St3gal5	Timp2	Rpl15	Atad2	Tuba1b
Sgk1	Cd74	Il18bp	Ccnd1	H2afx
Vsig4	Cbr2	Ssr4	Hells	Racgap1
Dusp1	Cx3cr1	Lgals3	Uhrf1	Cks2
Tnfaip2	Dab2	Trem2	Ptma	Tmpo
Cd5l	Ptafr	Rplp0	Topbp1	H2afv
Rasgef1b	Pira2	Nme2	Slbp	Cenpe
Gm47283	Ltc4s	Plac8	Dnmt1	Tpx2
Adam10	Gm21188	Ppib	Hat1	Ube2s
Cd44	Clec4n	Rpl12	Nasp	Nucks1
Dapk1	Prxl2b	Rps7	Hmgb2	Cdca3
Rsrp1	Rab3il1	Wfdc17	Ranbp1	Hmgn2
Rgs2	Ccr3	Cd5l	Rpa2	Ccna2
Klf2	Hspa1b	Tyrobp	Hnrnpab	Rrm2
Skil	Gfra2	Calr	Ran	Cdk1
Sowahc	Hspa1a	Ifitm3	Dek	Hmgb1
Sdc3	Ninj1	Ubb	Nap1l1	Lmnb1
Rcan1	Acvrl1	Rpl11	Dtl	Knstrn
Nr1h3	Gdf15	Rpl34	E2f1	Cenpf
St3gal4	Il16	Cox5b	Anp32b	Cks1b
Zbtb4	Gas6	Vim	Stmn1	Rrm1
Cebpb	Itm2b	Edf1	Fam111a	Smc4
2510009E07Rik	Wwp1	Rpl29	Cbx5	Rad21
Trim25	Tgfbi	Ndufa13	Slfn9	Tubb4b
Tent5a	Vamp5	Rpl28	Pclaf	Selenoh

C6	Fcgr3	Rpl10a	Gm47283	Cdca8
Rab32	Plbd1	Atp5g1	Dck	Ncapd2
Gbp2b	P2ry6	Rps15a	Dut	Atad2
Dock10	Pirb	Ctla2b	Alyref	Smc2
Cd163	Fcer1g	Rpl19	Hsp90aa1	Hmmr
Smad7	Tmsb10	Rpl18a	Nsd2	Ran
Pltp	Il18	Rps18	Set	Prc1
Tmem268	C5ar1	Rpl13	Gatm	Pcna
Tgm2	Mpp6	Rpl26	Fen1	Ptma
Grk2	Tgfb1	Ftl1	Gmnn	Kif11
Cybb	Fth1	Uqcrb	Usp1	Anp32e
Mtus1	Ap2a2	Cox8a	Tmpo	Tagln2
Lgmn	Hpgds	Cox5a	Wdhd1	Vim
Slc7a8	Lst1	Rpl10	Cdca7	Cdc20
Oas3	Mtss1	Clec1b	Tipin	Kif15
Mef2a	Irf2bp2	Ccl24	Hspd1	Plk1
Plac8	Scarb1	Rpl4	Ezh2	Tk1
Cers6	Malat1	Rpl18	Lmnb1	Nusap1
Cd207	Hgsnat	Manf	Chaf1a	Tacc3
Usp25	Clec4a2	Sec61b	H2afz	Anp32b
Egr1	Zmiz1	Blvrb	Dnajc9	Bub1b
Gadd45g	Pld3	Cd68	Clec4f	Kif20a
Apoc1	Adcy3	Prdx4	Naa50	Hnrnpa1
Sat1	Abcg3	Rps13	Srsf7	Ckap2l
Ifit2	Mcoln2	Rpl35	Ung	Ccnb1
Paqr9	C3	Rps19	Id2	Dek
Oasl2	Irf8	Rpl17	Mcm10	Jpt1
Ccnd1	Maf	Cyba	Serbp1	H2-Q7
Arl4c	Parvg	Rps16	Syce2	Cbx3
Rin2	Dhrs3	Cox7b	Dtymk	Spc24
Cd72	Fcna	Gpx3	Cct6a	Hp1bp3
Apobec1	Slc12a2	Rps6	Rbl1	Rangap1
Scd1	Enpp1	Ndufb8	Rrm2	Aurkb
Mpeg1	Cyba	Uba52	Srsf2	Asf1b
Slc15a3	Mt1	Pkm	Pole4	Ppia
Tcf7l2	Sh3gl1	Rpl21	Cgas	Ifi27l2a
Fos	Rgs10	Rps5	Top2a	Ckap2
Hsp90b1	Crip1	Rpl36al	Pa2g4	Mad2l1
Siglece	Glul	Cd52	Rpa1	Tuba1c
Frmd4a	Clec10a	Sec61g	Smc2	Mcm5
Dmwd	Cmah	Ifi27l2a	Rfc2	Incenp
Ptgis	Gna12	Sdf2l1	Hnrnpf	Ckap5
B4galt6	Tgfbr2	Atp5j	Dhfr	Anln

Pitpnc1	Sh3bgrl3	Ctsd	Hspa9	Hnrnpa3
Cd164	Mmp13	Psmb2	Fignl1	Dck
Stx3	Igfbp4	Rpl9	G3bp1	Ezh2
Nfam1	Ms4a6b	Rpl23	Cdh5	Cdkn2c
Nampt	Bcl2a1b	Rps10	Srsf3	Prdx4
Slc25a33	Gm34084	Rpl22l1	Cbx3	Kif2c
Ly6a	Fcgr2b	Cdh5	Mms22l	Kif20b
Adcy4	Lgals1	Abcc3	Cks1b	Ncaph2
Znfx1	Jup	Rps27a	Cdk4	Hist1h2ae
Klra2	C1qb	Tmed10	Ssrp1	Calm2
Btg1	Apobec3	Ldha	Nop56	Diaph3
Itm2c	Atp13a2	Rpl6	Snrpd1	Alyref
Slfn5	Snx6	Elob	Cd5l	Srsf3
Gm42418	Cd55	Hint1	Tagln2	Iqgap3
Cxcl13	Pycard	Eif5a	Gins2	Slfn9
Nfkbia	AC149090.1	Atp5b	Nucks1	Arhgap11a
Creg1	Pip4k2a	Slc15a3	Psip1	Mcm7
Ifi204	Pou2f2	H2-Aa	Fus	Cip2a
Tob2	Cxcl9	Il1b	Ptges3	Lbr
Soat1	Plxna1	Serinc3	Rbbp7	Cep55
Ifi211	Selplg	H2-Ab1	Casp8ap2	Knl1
Sema4c	Ccl9	Cd74	Cdk2	Arl6ip1
Ctss	Rps11	Selenop	Siva1	Pimreg
Slc40a1	Tmem51	Ms4a7	Eif5a	H3f3b
Cyth1	Gdpd1	Vsir	Baz1b	Aspm
Spop	Cyb5a	Clec2d	Paics	Hint1
Il1rn	Ptms	H2-Eb1	Pold1	Prr11
Ptgs1	Cd82	Dusp1	Id1	Lsm2
Smpdl3a	Cd81	Zfp36l2	Nrm	Plac8
Mndal	Camk1	Klf2	Cnbp	Hist1h4i
Cebpg	Rasa4	Irf2bp2	Npm1	Bub1
Dyrk2	Eno1	Spic	Ipo5	Ccdc34
Junb	Nrros	C4b	Pkmyt1	Lsm5
Itgav	Otulinl	Trf	Rbm3	Dbf4
Ddhd1	Asb2	Glul	Ncl	Foxm1
Xdh	Calm2	Fcgrt	Cenps	Anapc5
Adamdec1	Grk3	Gm47283	Suz12	Rbm3
Ncf1	Rras	Dab2	Timeless	Uhrf1
Engase	Cdk14	Rsrp1	Nup62	Nap1l1
Dusp3	Cbfa2t3	Junb	Hnrnpd	Spc25
Wwc2	Sulf2	Maf	St3gal5	Cdkn3
Clk1	Blvrb	Tsc22d3	Rif1	Kif22
Calhm2	Serpinb6a	Vcam1	Rfc3	H2-Q4

Cept1	Nav1	Stk17b	Zfp367	Snrpd1
Cd209f	Reep5	Ptprc	Atad5	Hmgn1
Ms4a7	Ciita	Pira2	BC005537	Smchd1
Vcam1	Snx5	C5ar1	Eif4a1	Dlgap5
Selenop	Tspo	Zfp36	Hnrnpa3	Rpsa
Cd209g	Ugcg	AC149090.1	Srm	Hdgf
Marco	Clec4a1	Klf6	Rbbp4	Lig1
Trf	Slc29a3	Timp2	Snu13	Hnrnpab
H2-Eb1	2410006H16Rik	Plbd1	Xist	Ubald2
Fcgrt	Psap	Zfhx3	Msr1	Hist1h1b
Spic	Marco	Ier5	Itgal	Sae1
Cx3cr1	Cyb5r1	Dusp6	Selenop	Cbx5
Cbr2	Tagln2	Il10ra	H2-Eb1	Melk
Vsir	Smagp	Dck	H2-Aa	Cxcl2
Hspa1b	Unc119	Jund	Ms4a7	Sfpq
Hspa1a	0610012G03Rik	Dhrs3	H2-Ab1	Fabp7
Timp2	Fam102b	Ptgis	Cd209f	Nuf2
Ninj1	Dnase1l3	Lair1	Cd74	Cbfb
Acvrl1	Ehd4	Cd83	Vcam1	Hnrnpa2b1
Serinc3	Alb	Cbr2	Trf	Rpl22l1
Cyba	Aplp2	mt-Nd3	Spic	Mis18bp1
Gm21188	Stard8	Klhl9	Fcgrt	Fbxo5
Gfra2	P2rx4	Pim1	Cd209g	Nrm
H2-Aa	Camk2d	Ptafr	Vsir	Usp1
Prxl2b	H2-DMa	Ddx3x	Fcgr3	Lockd
Rab3il1	Frmd4b	Fhod1	Hist1h1c	Cit
H2-Ab1	Cyth4	Btg1	Itm2b	Pbk
Il18	Inpp4a	Fam43a	Timp2	Kifc1
Clec4n	Comt	Egr1	2410006H16Rik	H2-Q6
Ptafr	Cd300a	Pirb	Pira2	Calm3
Dab2	Dstn	Adgre4	Serinc3	Gmnn
Ltc4s	Fxyd2	Trim25	Pirb	Tyms
Tmsb10	Cdc42ep2	Tmcc3	Lst1	Kif4
Tagln2	Abl1	Mrc1	Hist1h2bc	Snrpg
Eno1	Lair1	Marcks	Apoe	Rps19
Gdf15	Usp24	Wsb1	Fcer1g	Ndc80
Rps11	C1qa	Ndst1	H2-DMa	Ncapg
Ccr3	Ubb	Rhob	Cx3cr1	Dnmt1
Blvrb	Gm36161	Il6ra	Dab2	Id2
Fcer1g	Blnk	Psap	Acvrl1	Ccnf
Stmn1	Ms4a8a	Zfp703	Malat1	Ranbp1
C1qb	Hebp1	Prkcd	Maf	Ssrp1
Gapdh	Ptprc	Tgfbi	Cbr2	Shcbp1

Ubb	Pecam1	Txnip	Clec4n	Dynll1
Scarb1	Tmem176b	Ogt	Il16	AI506816
Clec4a2	Ndst1	Cd163	Ptafr	Rbbp7
Cd74	Rap1a	C6	Bcl2a1b	Aurka
Vamp5	Cd86	Laptm5	Rpl10	Mcm10
Fcgr3	Ggta1	Gatm	Mxd4	Nek2
Pira2	Ier3	Tab2	Slfn5	Smc1a
Sh3bgrl3	Herpud1	Pip4k2a	Tgfbi	Spag5
Mpp6	Adgrl3	Klf4	C1qb	Reep4
Hgsnat	Clec4f	Srsf5	Ccr3	Hnrnpu
Itm2b	Id3	Dazap2	Ubb	Pkmyt1
Rps3	Fabp7	Kcnj16	Clec4a2	Oxct1
Tgfb1	Id2	Mcl1	Gm21188	Snrpe
Snx6	Cdh5	Fos	Rgs10	Hsp90aa1
Il16	Il18bp	Ccr3	Hspa1b	Nsd2
Rpl15	Itgal		Hpgds	Rdm1
Pycard	Cd5l		Gas6	Neil3
Fth1	Id1		Irf2bp2	Mrfap1
Lst1	Helz2		Hist3h2a	Rps5
Alb	Gpx1		Prxl2b	Slbp
Pf4	Cd207		Blvrb	Cmc2
C3	Plac8		E230029C05Rik	Sgo1
Rpl32	St3gal5			Rps27l
Wwp1	BC005537			2410006H16Rik
Gas6	Dmpk			Brd8
Mt1	Vsig4			Xpo1
P2ry6	Cxcl13			Cenpw
Rpl21	Rab32			Arhgef39
Rps2	Rasgef1b			Gm42047
Hint1	Cd44			Fam111a
Tgfbi	St3gal4			Ncaph
Cox8a	Il1b			Ndc1
Rgs10	Ccnd1			Hist1h3d
0610012G03Rik	Sgk1			Hist1h1c
Cd68	Adam10			Nde1
Pld3	Rcan1			H1f0
Ftl1	Slc15a3			Sgo2a
Mcoln2	Tgm2			Atp5g2
Abcg3	Gm47283			Set
Enpp1	2510009E07Rik			Rad51
Cd55	Nr1h3			Cdc25b
Nme2	Tnfaip2			Snrpf
Clta	Tmem268			Cdca2

Cxcl9	Slc7a8			Topbp1
Cox5b	Sowahc			Rpl3
Rpl12	Dapk1			Dnajc9
Rpl29	Tmed10			Anapc11
Parvg	Apoc1			Hat1
Reep5	Cybb			Tardbp
Hpgds	Smad7			Gas2l3
Tmem37	Gadd45g			Dut
Ndufa13	Hsp90b1			Hist1h1d
Ppia	Skil			Prrc2a
Rpl10	Scd1			Pnp
Igfbp4	Pltp			Rad51ap1
Sh3gl1	Dock10			Nt5dc2
Rps10	Rgs2			Plk4
Atp13a2	Ptgs1			Prelid1
Gm34084	Cebpb			Hist1h1e
Mtss1	Zbtb4			Cenpq
Rps15a	Sdc3			Psip1
Clec10a	Hk3			Sun2
Ppib	Cers6			Rps20
Tspo	Oas3			Bub3
Lgals1	Clec1b			Kpna2
Cyb5a	Msr1			Smc3
Elob	Creg1			Nasp
Kcnq1ot1	Usp25			Nxt2
Comt	Gbp2b			Ect2
Calm2	Dmwd			Cxcl10
Ap2a2	Hk2			Ewsr1
Zmiz1	Ctla2b			E2f8
Tubb5	Wfdc17			Sms
Rpl10a	Sat1			Cenpm
Cd86	Slc25a33			Rfc5
Rpl18	Grk2			Ddx39
	Fam167a			Gm10076
	Hmgb2			Mcm3
	Lgmn			Tpm4
	Il1rn			Mrpl51
	Cd72			Fen1
	Tspan15			Srsf1
	Mtus1			Banf1
	Txnrd1			Zwilch
	Fgl2			Espl1
	Smpdl3a			Cpd

	Stx3			Selenop
	Rin2			Cd74
	Apobec1			Cd209f
	Cd164			Vcam1
	Emilin2			H2-Ab1
	Taldo1			Malat1
	Dusp1			H2-Aa
	Hic1			H2-Eb1
	Mpeg1			Fcgrt
	Acly			Ms4a7
	B4galt6			Trf
	Oasl2			Fcgr4
	Timd4			Hspa1a
	Pitpnc1			Il18bp
	Rsrp1			Cd209g
	Soat1			Kcna2
	Tent5a			Ltc4s
	Mef2a			Clec4n
	Nfam1			Lyz2
	Trim25			Ccnd1
	Lipa			Adgre1
	Pcolce2			Fcna
	Top1			Vsir
	Erp29			Itm2c
	Pdia6			Timp2
	Wwc2			Pitpnc1
	Nfkbia			Tbxas1
	Tk1			C6
	Siglece			Pirb
	Msrb1			Ninj1
	Sema4c			Hspa1b
	Ly6a			Cd81
	Frmd4a			Klf2
	Lrp1			Apoe
	Ifit2			AC149090.1
	Paqr9			Itm2b
	Cd163			Plbd1
	Hspa5			Pira2
	Stmn1			Ifitm2
	Psma6			Ctla2b
	Ppdpf			Crip2
	Cmas			Ucp2
	Tbc1d1			Klf4

	Dck			Pld3
	Surf4			Pilra
	Cyth1			Zbtb20
	Naa50			Csf1r
	Pabpc1			Ctsb
	Fam129a			Axl
	Nampt			Pilrb2
	Hnrnpa2b1			Gfra2
	Folr2			Psap
	Trem2			Siglece
	Gatm			Mrc1
	C6			Rhob
	Mcm5			Frmd4b
	Pgd			C4b
	Ifitm6			C1qa
	Znfx1			Spic
	Fam120a			Pou2f2
	Rpl41			Fcer1g
	Itga9			Cd163
	Coro2a			Pilrb1
	Ssr3			Glul
	Spop			Tmem86a
	Ptma			Ndfip1
	Ckap4			Bank1
	Ifi211			Jund
	Lpl			Dusp6
	Arl4c			Ptprj
	Cept1			Abcg3
	Rnf213			Bcl2a1b
	Pld1			Ctss
	Klf2			Mpeg1
	Dusp3			Camk1d
	Ncf1			Lpl
	Csf2rb			Lrp1
	Filip1l			Clec2d
	Gm10076			Apoc1
	Plod1			Prxl2b
	Ptgis			Sdc3
	Prdx5			Map1lc3a
	Tubb5			Tgfbr2
	Slc40a1			Tmcc3
	Tmed2			Marcksl1
	Itm2c			Acvrl1

	Itgav			C5ar1
	Adcy4			Cyba
	Tcf7l2			Junb
	Adam17			Txnip
	Rnpep			Slfn5
	Calhm2			Cd83
	Nlrp3			Ptafr
	P4hb			Cd14
				Kctd12
				E230029C05Rik
				Rabac1
				Pik3ap1
				Slc40a1
				Arf3
				Lair1
				Trpm2
				Fam43a
				Cyth4
				Pld4
				Pecam1
				Slc12a2
				Lbh
				Samhd1
				Lgmn
				Ccl6
				Hdac9
				Ccr3
				Atp13a2
				Timd4
				Ckb
				Vav2
				Pla2g7
				Ap2a2
				Lilrb4a
				App
				Tyrobp
				Asah1
				Fth1
				Fcgr3
				Dhrs3
				Gns
				Pltp
				B430306N03Rik

				Pepd

Supplemental Table 2. Antibodies used for flow cytometry.

Antibodies	Company	Catalog#
CD16/CD32 (clone 93)	eBioscience	14-0161-85
CD45_AF700 (clone 30-F11)	BD Bioscience	560510
CD45_redFluor 710 (clone 30-F11)	TONBO Bioscience	80-0451
CD45_FITC (clone 30-F11)	TONBO Bioscience	35-0451
CD115_PE (Clone 12-3A3-1B10)	eBioscience	12-1159-42
CD115_PE-Cy7 (clone AFS98)	Biolegend	135523
CD11b_PE-Cy7 (clone M1/70)	eBioscience	25-0112-82
CD11b_AF700 (clone M1/70)	Biolegend	101222
CD11c_eFluor 660 (clone N418)	eBioscience	50-0114-80
CD11c_PE (clone N418)	TONBO Bioscience	50-0114-U025
CD11c_ PE-Cy7 (clone N418)	Biolegend	117317
Alexa Fluor 647 donkey anti-goat	Invitrogen	A-21447
CD45.1_BV421 (clone A20)	Biolegend	110731
CD45.2_PE (clone 104)	Invitrogen	12-0454-82
CD45.2_AF700 (clone 104)	Biolegend	109822
CD64_APC (clone x54-5/7.1)	Biolegend	139306
CD64_PE (clone x54-5/7.1)	Biolegend	139303
CD64_BV421 (clone x54-5/7.1)	Biolegend	139309
CLEC4F (Polyclonal)	R&D SYSTEMS	AF2784
F4/80_Super Bright645 (clone BM8)	eBioscience	64-4801-82
F4/80_PE (clone BM8)	Invitrogen	MF48004,
F4/80_AF647 (clone BM8)	Biolegend	123122
Ly6C_PE (clone HK1.4)	eBioscience	12-5932-80
Ly6C_Alexa Fluor 488 (clone HK1.4)	Invitrogen	53-5932-80
Ly6C_PE-Cy7 (clone HK1.4)	Biolegend	128017
Ly6G_PE-Cy7 (clone 1A8)	BD Bioscience	560601
Ly6G_Bv421 (clone 1A8)	BD Bioscience	562737
MHC-Ⅱ_eFlour 450 (clone M5/114.15.2)	eBioscience	48-5321-82
SiglecF_BV421 (clone E50-2440)	BD Bioscience	562681
Tim4_PE (clone 54(RMT4-54))	eBioscience	12-5866-82
Tim4_APC (clone 54(RMT4-54))	TONBO Bioscience	20-5866
Tim4_AF647 (clone 54(RMT4-54))	Biolegend	130008
VSIG4_PE (clone NLA14)	eBioscience	12-5752-82
VSIG4_APC (clone NLA14)	eBioscience	17-5752-80
CD317_APC (clone 927)	Biolegend	127015
IFN-γ_PE (clone XMG1.2)	eBioscience	12-7311-82
CD8α_ violetFluor 450 (clone 53-6.7)	TONBO Bioscience	75-0081
CD3_FITC (clone 17A2)	Biolegend	100203
CD3_APC (clone 17A2)	TONBO Bioscience	20-0032
CD4_FITC (clone RM4-5)	eBioscience	11-0042-81

CD19_FITC (clone 1D3/CD19)	Biolegend	152403
NK1.1_FITC (clone PK136)	Biolegend	108705
TCR gamma/delta_PE (eBioGL3)	eBioscience	12-5711-82
IL-17A_eFluor 450 (eBio17B7)	Invitrogen	48-7177-82

Supplemental Table 3. Primers used for real-time PCR.

Gene	Forward primer	Reverse primer
Pol2	CTGGTCCTTCGAATCCGCATC	GCTCGATACCCTGCAGGGTCA
Alk1	GGCCTTTTGATGCTGTCG	ATGACCCCTGGCAGAATG
Alk2	CACCACCAATGTGGGAGACA	TTCCCGACACACTCCAACAG
Alk3	ACACTGCCCAGATGATGCTA	GGAAATGAGCACAACCAGCC
Tgfbr2	TAACAGTGATGTCATGGCCAGCG	AGACTTCATGCGGCTTCTCACAGA
Smad4	CCTGTTGTGACTGTGGATGGCTATG	AGACCTTTATATACGCGCTTGGGTAGA
Id1	CTCAGCACCCTGAACGGCGA	CATCTGGTCCCTCAGTGCGCC
Id3	CACTTACCCTGAACTCAACGCC	CCCATTCTCGGAAAAGCCAG
Clec4f	CTTCGGGGAAGCAACAACTC	CAAGCAACTGCACCAGAGAAC
Fabp7	GAGACAAGCTCATTCATGTGCAG	ACTCCAGGAAACCAAGTTGTC
Cd5l	GAGGACACATGGATGGAATGT	ACCCTTGTGTAGCACCTCCA
Cdh5	TCAACGCATCTGTGCCAGAGA	CACGATTTGGTACAAGACAGTG
Vsig4	TCATTGAGCTCCGTGTTCGG	GGAGTGCAGGGTTGTAGGTG
Tnfα	TCCAGGCGGTGCCTATGT	CACCCCGAAGTTCAGTAGACAGA
Mcp1	TAAAAACCTGGATCGGAACCAAA	GCATTAGCTTCAGATTTACGGGT

Full unedited gel for Supplemental Figure 5C

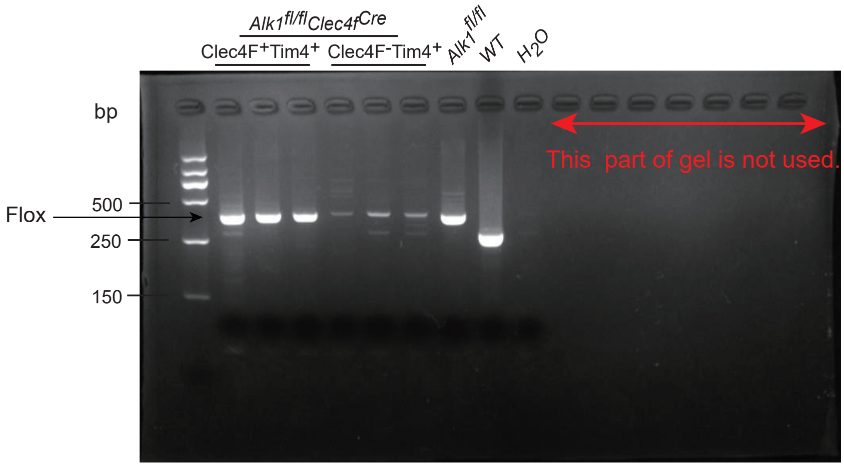

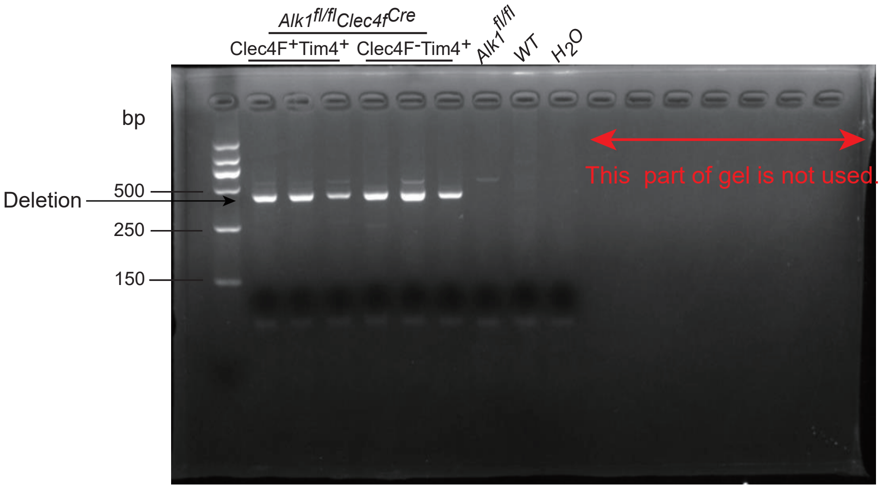

Supplement: Supplemental data [file jci-132-150489-s022.pdf]
